# Ecology of Subseafloor Crustal Biofilms

**DOI:** 10.3389/fmicb.2019.01983

**Published:** 2019-08-28

**Authors:** Gustavo A. Ramírez, Arkadiy I. Garber, Aurélien Lecoeuvre, Timothy D’Angelo, C. Geoffrey Wheat, Beth N. Orcutt

**Affiliations:** ^1^Graduate School of Oceanography, University of Rhode Island, Narragansett, RI, United States; ^2^Division of Biological Sciences, University of Montana, Missoula, MT, United States; ^3^Bigelow Laboratory for Ocean Sciences, East Boothbay, ME, United States; ^4^Université de Bretagne Occidentale, UFR Sciences et Techniques, Brest, France; ^5^Institute of Marine Science, University of Alaska Fairbanks, Fairbanks, AK, United States

**Keywords:** deep biosphere, subseafloor, oceanic crust, Juan de Fuca, CORK, FLOCS, low biomass

## Abstract

The crustal subseafloor is the least explored and largest biome on Earth. Interrogating crustal life is difficult due to habitat inaccessibility, low-biomass and contamination challenges. Subseafloor observatories have facilitated the study of planktonic life in crustal aquifers, however, studies of life in crust-attached biofilms are rare. Here, we investigate biofilms grown on various minerals at different temperatures over 1–6 years at subseafloor observatories in the Eastern Pacific. To mitigate potential sequence contamination, we developed a new bioinformatics tool – *TaxonSluice*. We explore ecological factors driving community structure and potential function of biofilms by comparing our sequence data to previous amplicon and metagenomic surveys of this habitat. We reveal that biofilm community structure is driven by temperature rather than minerology, and that rare planktonic lineages colonize the crustal biofilms. Based on 16S rRNA gene overlap, we partition metagenome assembled genomes into planktonic and biofilm fractions and suggest that there are functional differences between these community types, emphasizing the need to separately examine each to accurately describe subseafloor microbe-rock-fluid processes. Lastly, we report that some rare lineages present in our warm and anoxic study site are also found in cold and oxic crustal fluids in the Mid-Atlantic Ridge, suggesting global crustal biogeography patterns.

## Introduction

Sediment-buried oceanic crust is one of the most inaccessible and understudied habitats on Earth (Orcutt et al., 2011b; Edwards et al., 2012). Upper oceanic crust, with an estimated volume of 10^18^ cubic meters (Fisk et al., 1998), hosts a global subseafloor aquifer (Fisher and Becker, 2000) that cycles through the crust at a water volume-equivalent to that of the planet’s oceans every 10^5^ to 10^6^ years (Elderfield and Schultz, 1996; Wheat et al., 2003). This creates a water-rock thermodynamic disequilibrium that may power chemolithoautotrophy (Bach and Edwards, 2003; Edwards et al., 2005). The large volume of this habitat, despite lower biomass loads compared to overlying sediments (Kallmeyer et al., 2012), makes relatively small contributions of *in situ* dark autotrophy potentially significant to the local and global carbon cycle (McCarthy et al., 2010; Edwards, 2011; Orcutt et al., 2015).

A major challenge to ecological studies of subseafloor crust is containing and mitigating inadvertent contamination of low biomass samples during seafloor drilling (Lever et al., 2006, 2013; Masui et al., 2008; Santelli et al., 2010; Labonte et al., 2017). To this end, subseafloor observatories called CORKs (Davis et al., 1992; Wheat et al., 2010) were used successfully in microbiological studies of crustal fluids at the eastern flank of the Juan de Fuca (JdF) Ridge (Cowen et al., 2003; Jungbluth et al., 2013, 2014, 2016, 2017a; Robador et al., 2014) and at North Pond (NP) on the western flank of the Mid-Atlantic Ridge (Meyer et al., 2016; Shah Walter et al., 2018; Tully et al., 2018). These studies show that plankton communities in warm and anoxic JdF crustal fluids change over time and are comprised of unique lineages within the Firmicutes, Deltaproteobacteria, Aminicenantes/OP8, and Thermotogae, that are rare in other marine systems. In contrast, the cool and oxic crustal plankton communities at NP are enriched in Gamma- and Epsilon-proteobacteria and are more similar in structure to bottom seawater. Recent metagenomic analysis suggests that NP crustal fluid planktonic communities are functionally stable despite temporal shifts in dominant taxonomic groups (Tully et al., 2018).

Despite progress in the study of crustal fluid plankton, the nature of crust-attached biofilms in this habitat is less well understood. Mineral colonization experiments using Flow-through Osmotic Colonization Systems [FLOCS, (Orcutt et al., 2010)] deployed within CORK observatories have grown biofilms *in situ* from crustal fluid inoculant (Orcutt et al., 2011a; Smith et al., 2011, 2016; Baquiran et al., 2016). These studies suggest that FLOCS-grown biofilms (contaminant-free proxies of natural crustal biofilms) are more similar to each other than the surrounding fluids seeding them, suggesting structural and potentially functional distinctions between crustal biofilm and plankton. The driving environmental factors and ecological effects of these differences are unknown. To our knowledge, metagenomic analysis of native subseafloor crustal biofilm communities has not be successful to date, although there are two recent metagenomic assessments of biofilms formed during incubations with subseafloor rocks *in vitro* (Zhang et al., 2016) and *in situ* (Smith et al., 2019). These studies suggest potential for carbon fixation by biofilm communities using the Wood-Ljundahl reductive TCA cycle, and also the potential for dissimilatory nitrate reduction. In oxic *in vitro* incubations of subseafloor basalts, iron cycling pathways appear to be prevalent (Zhang et al., 2016) whereas in an *in situ* iron-rich olivine incubation within anoxic sediment buried crust, sulfate reduction is more prevalent (Smith et al., 2019). Prior studies suggest that mineralogy is a driving factor influencing biofilm community structure on seafloor and subseafloor minerals (Toner et al., 2013; Smith et al., 2016), and one study also suggests that temperature is another important factor (Baquiran et al., 2016).

**FIGURE 1:**
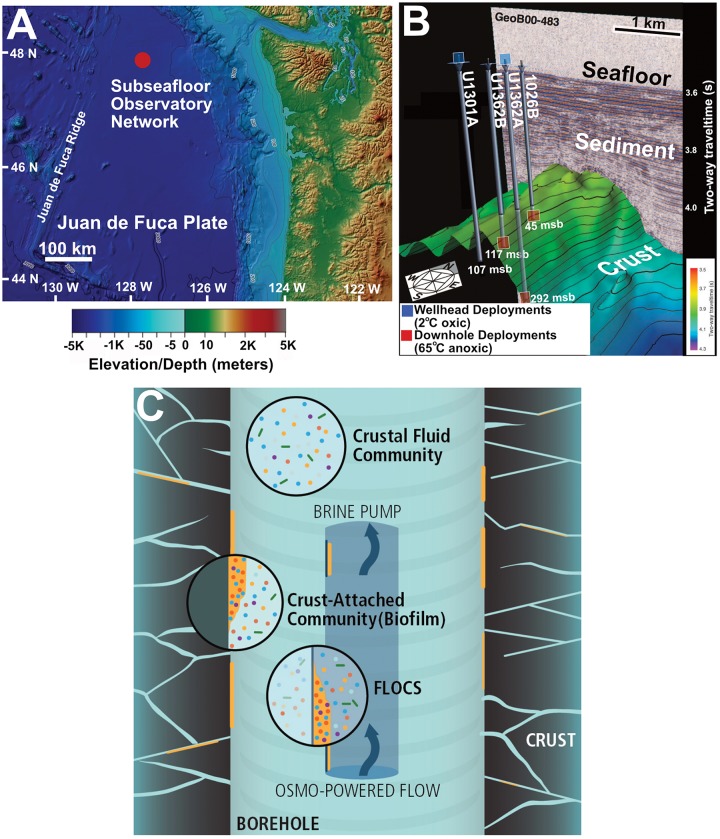
**(A)** Location of Juan de Fuca Ridge CORK observatory sites in the northeastern Pacific Ocean. **(B)** Overview of subsurface crustal topography and penetration of CORK observatories used for experiment deployment in this study. IODP originated figure modified from Expedition 327 Scientists (2011b). **(C)** Schematic of FLOCS *in situ* deployment highlighting the differences in planktonic and crust-attached communities.

To further examine the nature of biofilms in the subseafloor crust, we assess biofilm community response to different environmental conditions through microbe-mineral incubation experiments within CORKs at the JdF Ridge flank (Figure 1). The incubations varied in mineral substrates, deployment temperature, and time, but all accessed the same subsurface crustal aquifer fluids. 16S rRNA gene amplicon sequence analysis of these low-biomass samples required the development of a new bioinformatics tool – *TaxonSluice* – for robust elimination of potential contaminant sequences. By comparing biofilm amplicon datasets recovered from our incubations to previously published metagenomic datasets from JdF and NP crustal fluids, as well as a metagenome from a subseafloor incubation of olivine at JdF, genetic differences with respect to potential activities of crust-attached biofilm and planktonic communities are assessed and the extent of biogeographical connectivity of the subseafloor crust is explored.

## Materials and Methods

### Study Site

The four JdF subseafloor observatories targeted in this study include a transect of cased boreholes with CORKs that penetrate into ∼3.5 Ma year-old sediment-covered basaltic crust along a fluid flow path (Figure 1 and Supplementary Table S1), as described elsewhere (Expedition 327 Scientists, 2011a). This configuration allows subseafloor conditions to return to a pre-drilling hydrogeological state by stopping bottom water intake (Fisher et al., 2011). Two observatories in the transect (Holes 1026B and U1301A) were previously used for mineral incubation studies (Orcutt et al., 2011a; Smith et al., 2011; Baquiran et al., 2016). The other two observatories, with upgraded materials to prevent corrosion, were installed during Integrated Ocean Drilling Program (IODP) Expedition 327 in 2010 (Fisher et al., 2011; Jungbluth et al., 2016). Overall, fluid conditions in the subsurface crust at this site are characterized as warm (64°C), anoxic, and sulfate-replete (Wheat et al., 2011; Neira et al., 2016).

### Experiment Design

Colonization experiments were deployed at depth in the observatories during IODP Expedition 327 in 2010 and at the observatory wellheads on cruise AT26-03 in 2013 (Figure 1B and Supplementary Table S2). All samples were recovered in August 2014 during cruise AT26-18 of the R/V *Atlantis* (Woods Hole Oceanographic Institution). FLOCS deployed at depth downhole were exposed to *in situ* conditions within the anoxic 64°C crustal subseafloor aquifer, whereas FLOCS deployed at the wellhead are inoculated with the same crustal fluid sourced from below but are chilled in the surrounding 2°C oxic bottom water at the seafloor (Figure 1B and Supplementary Table S1). Wellhead FLOCS conditions are therefore cold and may have trace levels of oxygen from diffusion across FLOCS plastic materials, as discussed elsewhere (Baquiran et al., 2016). Briefly, FLOCS used in this study consisted of sterile massive basalts (previously cored from JdF Holes 1027B/U1301B) and commercially available pyrite (FeS_2_, Ward’s Science, Catalogue 466448) crushed (<250 μm grain size) and autoclaved prior to deployment as described elsewhere (Fisher et al., 2011). For additional details on FLOCS deployment configuration and recovery, see Supplementary Information.

### Microscopy, Geochemical, and DNA Analyses

Methods used for sample preservation, scanning electron microscopy (SEM), geochemical analyses of the fluids exiting the FLOCS units, DNA extraction, quantitative polymerase chain reaction analysis of the small subunit ribosomal RNA (16S rRNA) gene, 16S rRNA gene amplicon sequencing via Illumina, and data processing steps are described in detail in the Supplementary Material. Due to low DNA concentrations in the extracts (Supplementary Table S3), replicate extracts from similar experiments and minerals were pooled according to sample identity and further concentrated prior to 16S rRNA gene amplification. Extracts from procedural blanks were also pooled and sequenced to address2 inadvertent contamination, as described below. Sequence data was processed with *mothur* (Schloss et al., 2009) at the 97% or greater sequence similarity level defining an Operational Taxonomic Unit (OTU). All new sequence data from this study are publicly available in the NCBI Short Read Archive under BioProject PRJNA472057; BioSample accession numbers: SAMN09228041–SAMN09228055.

### Correcting for Potential Contaminant 16S rRNA Gene Sequences: *TaxonSluice*

As the amount of material available for analysis was limited, and the incubation settings are low biomass environments [*i.e.*: roughly 10^4^ cells ml^–1^ (Jungbluth et al., 2013)], significant effort was invested in evaluating protocol blanks to assess for possible contamination in the sequence data, as recommended elsewhere (Sheik et al., 2018). To identify contaminant sequences in low-biomass samples, we developed a new bioinformatic tool: *TaxonSluice*. The code, sample datasets (data used for this study), validation efforts on mock communities, and additional user information (dependencies and user-set parameters) for this tool are publicly available on GitHub^1^. Briefly, *TaxonSluice* implements a novel heuristic algorithm (Supplementary Material and Supplementary Figure S1) with OTU tables and user input to specify blanks to identify potential contaminant sequences based on a user-defined abundance threshold (10% default). In this study, extraction blanks were incorporated in each batch of sample extractions, enabling assessment of multiple blanks across the dataset. Algorithm outputs include a summary of all “flagged” OTUs (Supplementary Figure S1) along with percent identity, percent coverage, *e*-value and environmental source for the 10 closest matches in the SILVA database. The user validates output prior to culling any of the flagged OTUs from the data. This multiple-blank approach is advantageous because each blank represents independent biological data, outside the single sample and sample-specific blank couple.

### Linking Biofilm Amplicons and Metagenomic Data

To assess taxonomic overlap between the biofilm communities in this study with crustal fluid communities and other biofilm studies, biofilm 16S rRNA gene amplicon sequences recovered from our FLOCS incubations were used to recruit metagenomic reads from crustal fluids from JdF (Jungbluth et al., 2017a) and NP (Tully et al., 2018) as well as from a subseafloor incubation of olivine at JdF (Smith et al., 2019). Thus, we highlight that differentiation of biofilm and planktonic cells in these comparisons is exclusively done *in silico.* These publicly available metagenomes were downloaded from the NCBI Sequence Read Archive: JdF crustal fluid (NCBI BioSamples SAMNO3166137 and SAMO3166138 for Hole U1362A and U1362B fluids, respectively), NP crustal fluid [NCBI BioSamples SAMN07571231–SAMN07571245 for Holes U1382A and U1383C at various sampling times and depths, see Tully et al. (2018)], and JdF olivine [NCBI BioProject number PRJNA264811 (Smith et al., 2019)]. Metagenomes were assembled into contigs and binned into metagenome-assembled genomes (MAGs) following a pipeline described in the Supplementary Information.

**FIGURE 2:**
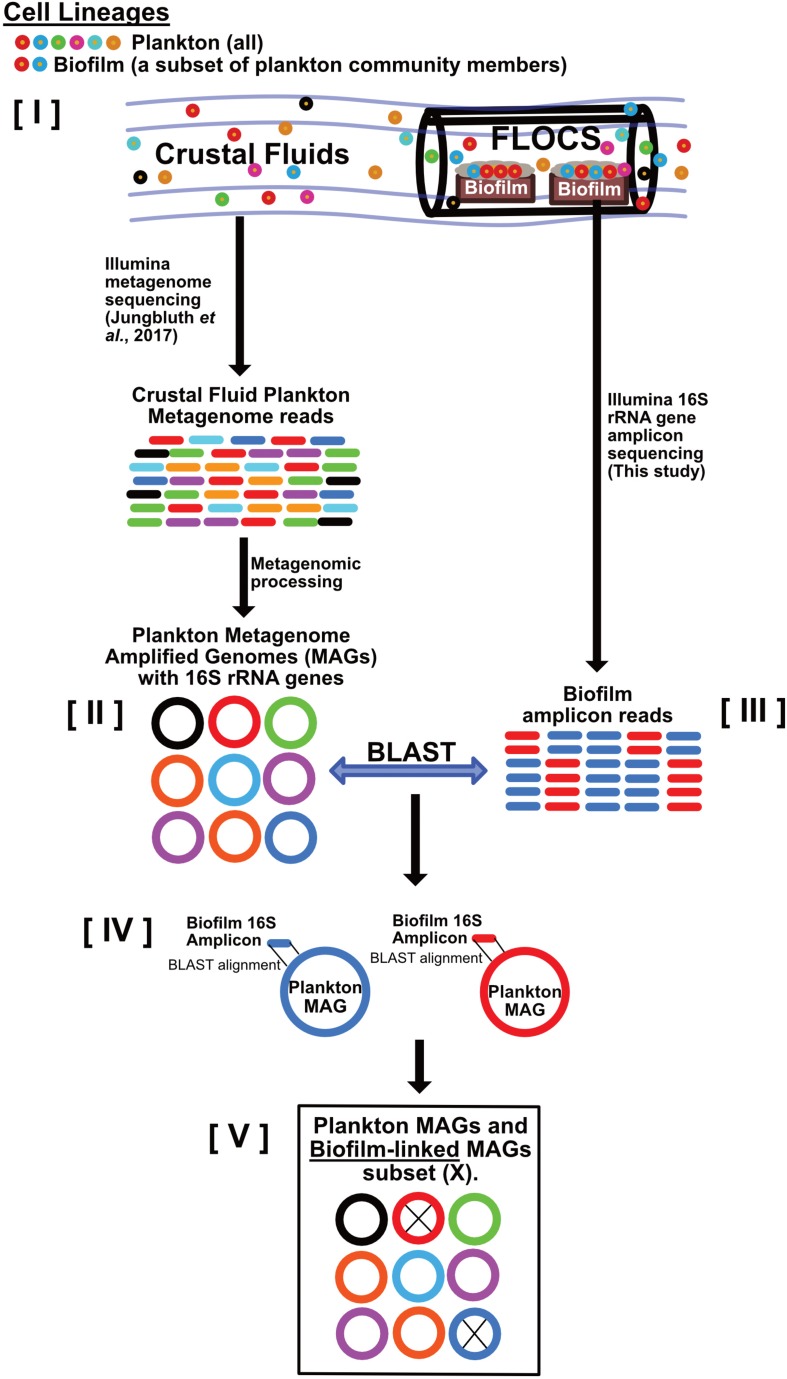
Schematic depicting our strategy for linking crustal fluid plankton MAGs to biofilm lineages. This workflow begins with the assumption that **[I]** all biofilm community members are also part of the crustal fluid community because FLOCS biofilms arise from crustal fluid inoculation. Metagenomic data from crustal fluid is processed into MAGs representing planktonic lineages **[II]**. Amplicons (16S rRNA gene) sequences from FLOCS biofilm **[III]** also represent planktonic lineages; however, these lineages transitioned from planktonic to biofilm lifestyles. Stringent local alignments **[IV]** between biofilm amplicons and the subset of plankton MAGs containing 16S rRNA genes reveal which planktonic lineages also reside in FLOCS biofilm and are thus considered “biofilm-linked” **[V]**.

To link functional potential of reconstructed MAGs to our FLOCS biofilms, 16S rRNA gene amplicons generated from biofilms were queried against the subset of MAGs containing full-length or near full-length 16S rRNA genes using BLASTn v2.2.30 + (Altschul et al., 1990), with the following parameters: -qcov_hsp_perc 0.9 -perc_identity 99.0. Crustal fluid MAGs with 16S rRNA genes meeting this alignment threshold were considered “biofilm-linked” (i.e., they are hypothesized to represent planktonic groups, or closest relatives, that transitioned from planktonic to biofilm lifestyles, see Figure 2). MAGs that had 16S rRNA genes not matching any of the FLOCS biofilm 16S rRNA gene amplicons were considered “planktonic”. We note that 16S rRNA genes are notoriously difficult to reconstruct from metagenomic data (Yuan et al., 2015) and consequently, not all of the reconstructed MAGs had them. To minimize biases, MAGs lacking 16S rRNA gene sequences were excluded from subsequent genomic comparisons of planktonic vs. biofilm-linked fractions. For quality, assembly of 16S rRNA genes was assessed by mapping metagenome reads to the reconstructed 16S rRNA sequences using Bowtie2 v2.3.4.1 (Langmead and Salzberg, 2012), and manually examining the uniformity of read coverage. We also compared the overall read coverage among each reconstructed 16S rRNA gene and the contig from which each gene was predicted. MAGs with 16S rRNA genes with highly variable gene coverage profiles were excluded from all downstream analyses. MAGs were annotated using GhostKOALA (Kanehisa et al., 2016b). KEGG (Kanehisa et al., 2016a) assignments for each set of MAGs (biofilm-linked vs. planktonic) were normalized to total number of predicted ORFs and the resulting gene counts compared. For estimation of metabolic potential, a library of custom and publically available (Anantharaman et al., 2016) hidden Markov models (HMMs) was queried against each set of MAGs. The HMM library and associated scripts are available in GitHub: https://github.com/Arkadiy-Garber/taxonsluice/tree/master/custom_scripts_and_libs.

## Results

### Summary of Deployment Conditions and Chemical Analyses

FLOCS were deployed for periods of one and 4 years (Table 1). Despite the different locations, temporal records of dissolved magnesium and sulfur (sulfate) indicate that subsurface crustal fluids were pulled into all of the FLOCS chambers (Supplementary Figure S2). After an initial period of mixing of the crustal fluid with the sterile deployment solutions, the concentrations of these ions reach concentrations that are expected for this environment (Wheat et al., 2010). These chemical records confirm the structural and functional integrity of our experiments throughout the deployment periods and indicate that intrusion of bottom seawater did not occur.

**TABLE 1:** Presence of genes involved in various metabolic pathways in the biofilm and planktonic categories constructed from crustal fluid metagenome from the Juan de Fuca crustal subsurface (from Jungbluth et al., 2017a).

***Metabolism***	**Function**	**Gene**	**Biofilm**	**Plankton**
*Carbon fixation*	Wood Ljungdahl	codhCD	✓	✓
	Calvin cycle	Rubisco		✓
*Nitrogen*	Nitrate assimilation	NasA	✓	✓
	Nitrate reduction	NapAB	✓	✓
	Nitrate reduction	NarGH	✓	✓
	Dissimilatory nitrite reduction	NirBD	✓	
	Denitrification	NirS	✓	
	Nitrite reduction	NrfH	✓	
	Nitric oxide reduction	NorB	✓	✓
	Nitrous oxide reduction	NosZD		✓
*Sulfur*	Sulfate reduction	SopT	✓	✓
	Sulfate reduction	ApsK	✓	✓
	Sulfate reduction	CysN	✓	
	Sulfate reduction	AprA	✓	
	Sulfite reduction	AsrB		✓
	Sulfite reduction	dsrABCMK/dsrD	✓	
	Sulfur oxidation	dsrEFHCMK		✓
	Thiosulfate oxidation	SoxYZ	✓	
*Iron*	Iron reduction/oxidation	MtrA	✓	
*Hydrogen*	Hydrogen oxidation	Hydrogenases (various)	✓	✓
*Oxygen*	Oxygen reduction	Various	✓	✓
*CO*	Carbon monoxide oxidation	CO dehydrogenase	✓	✓

### Microscopic Evidence of Microbial Colonization

**FIGURE 3:**
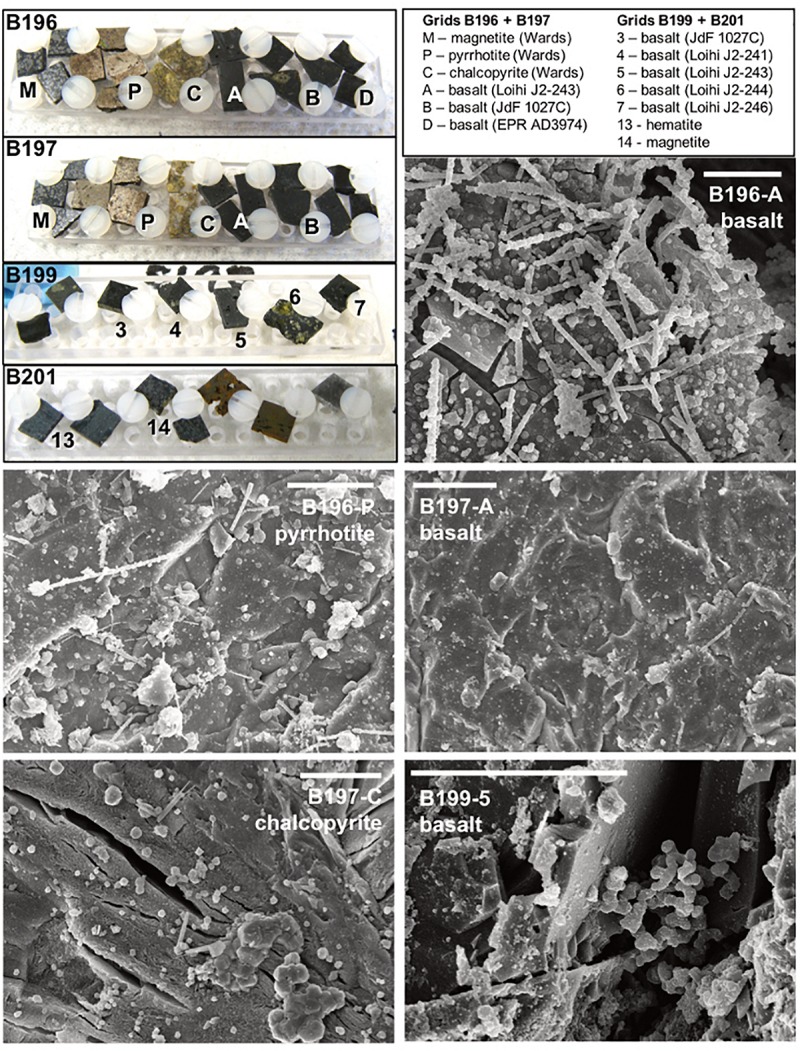
Select scanning electron micrographs (SEM) from rock chips incubated downhole at Hole U1362B (see Supplementary Figures S3–S6 for additional images). The upper left panel shows photographs of the rock chips before deployment on IODP Exp. 327, with grid labels and rock chip types indicated in the legend. Basalt chips were created from field samples collected previously from the East Pacific Rise (EPR, a glassy basalt collected on Alvin dive 3974), from Juan de Fuca Ridge flank (JdF, from ODP Hole 1027C), and from the Loihi Seamount (two highly vesicular aphyric basalts from ROV Jason dives J2-241 and J2-243, and two olivine-phyric basalt from dives J2-244 and J2-246). The metal sulfides chips were prepared from commercially available samples from Wards Geology. Representative scanning electron microscopy (SEM) micrographs from three basalt samples and two metal sulfides are shown; scale bars in each are 10 μm.

SEM confirmed mineral colonization and was primarily useful for looking at secondary mineral precipitates (Figure 3, and Supplementary Figures S3–S6), which can have characteristic shapes (Toner et al., 2009) in this environment. Cell morphologies observed included straight, sheath-like stalks (some with external encrustation), coccoid shaped cells, and some structures that resembled “Y” shaped stalked cells. The composition and origin of the sheath features is unknown, but morphologically they resemble stalks known to be made by some microaerophilic iron oxidizing bacteria (Barco et al., 2017). Notably absent are twisted stalks enriched in iron oxyhydroxides previously observed in JdF mineral colonization experiments that had been exposed to bottom seawater (Orcutt et al., 2011a).

### DNA Extraction, Gene Quantification, Sequencing, and OTU Quality Filtering

Bacterial 16S rRNA gene abundance varied from 2 × 10^2^ to 1 × 10^6^ gene copies per gram of rock; however, some samples were below the detection limit (Supplementary Table S3). The highest 16S rRNA gene abundances were observed on basalts enriched at wellheads. Illumina 16S rRNA gene sequencing resulted in 791,077 high quality sequences that were clustered into OTUs (Supplementary Table S3). *TaxonSluice* flagged 22 OTUs as potential sequence contaminants from kits. Ultimately, after assessing the *TaxonSluice* output, 17 of these 22 suspects were culled from further analyses. In sum, after implementing *TaxonSluice*, 401 Bacterial OTUs, representing 69.9% of the initial high-quality sequences, were further characterized.

### Bacterial Community Composition, Diversity and Structure

**FIGURE 4:**
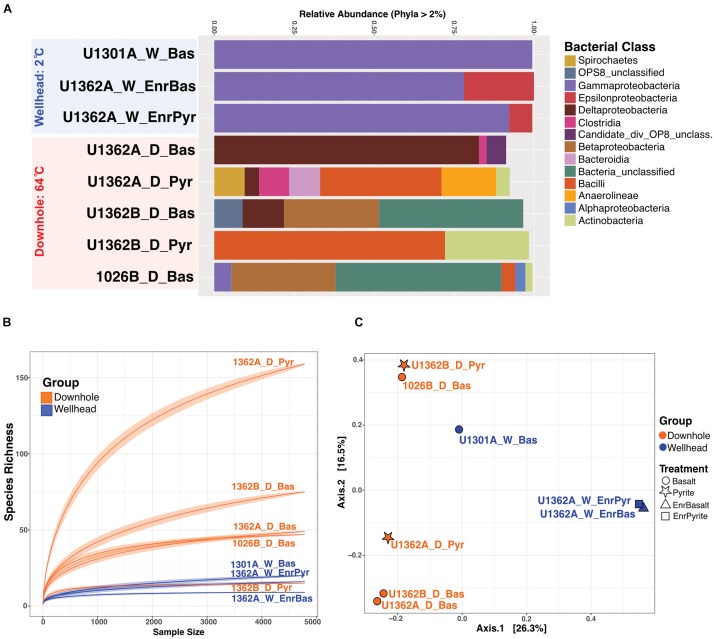
Overview of the 16S rRNA gene diversity in rock chip biofilms from Juan de Fuca CORK subseafloor observatories FLOCS experiments. **(A)** Relative abundance of 16S rRNA gene sequences in various bacterial groups (at the class level as shown in legend, for groups >2% of sequences) for the wellhead (blue) and downhole (red) FLOCS samples. **(B)** Rarefaction analysis of the same sequence data reveals the greater diversity in most of the downhole biofilms. **(C)** Principal Coordinate Analysis (PCoA) of the same sequence data grouped by deployment location (color) and treatment type (symbols; as shown in the legend) reveals the clustering of microbial communities based on deployment location.

Communities recovered from downhole incubations had more diverse taxonomic groups (Phylum-level) than those observed from wellhead deployments (Figure 4). While this may reflect the longer incubation time of the downhole incubations (Supplementary Table S2), we note that the DNA extracts were pooled from multiple samples and, therefore, cannot assess potential inter-sample variability. We thus interpret our community diversity survey as a qualitative rather than quantitative assessment. Proteobacteria comprised between 5–98% of the community of all incubations. All wellhead sequences grouped within Gamma- and Epsilon-proteobacteria. Firmicutes, Deltaproteobacteria, Aminicenantes (OP8), Thermotogae, Deferribacteres, Actinobacteria, Chloroflexi, Bacteroidetes, and Spirochetes were recovered from downhole 64°C anoxic incubations. The temperature-driven differential enrichment of Gamma- and Epsilon-proteobacterial lineages in wellhead deployments from warm the downhole crustal fluid communities is significant (Wald Test, *P*_*val*_ = 0.01, Supplementary Figure S12). Ordination patterns show communities recovered from different mineral substrates as similar (*e.g.*: 1362A_W_EnrBas associating with 1301A_W_Bas and 1362B_D_Pyr associating with 1026B_D_Bas; Figure 4C and Supplementary Figure S13). The first axis PCoA separated wellhead from downhole enrichments, implying that incubation environment (cool wellhead vs. warm downhole), rather than mineralogy, defined community structure.

### Phylogenic Comparisons

**FIGURE 5:**
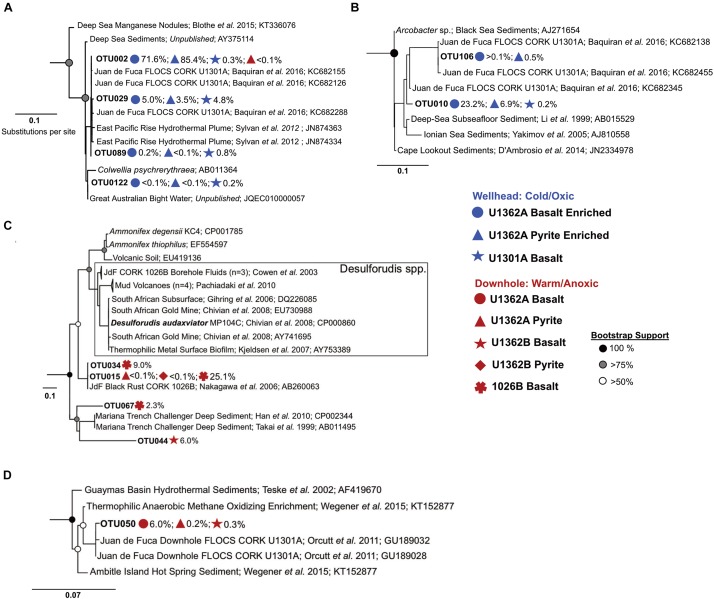
Maximum likelihood phylogenetic trees of abundant 16S rRNA gene OTUs recovered in this study compared to close environmental relatives (based on 100 bootstrap replications) show different dominant groups between the wellhead and downhole rock chip incubations. **(A)** Gammaproteobacteria tree. **(B)** Deltaproteobacteria tree. **(C)** Firmicutes tree. **(D)** Aminicenantes (candidate phylum OP8) tree. Colored icons show FLOCS deployment site and substrate for each recovered OTU portrayed. Gray scale circles show bootstrap support per branch. Scale bars indicate substitutions per site. More detailed trees provided in Supplementary Figures S7–S9.

There was nearly no overlap between OTUs from cold (2°C) wellhead and warm (64°C) downhole incubations (Figure 5 and Supplementary Figures S7–S9). Phylogenetic analysis of our amplicons does not indicate matches to bottom seawater groups (Supplementary Figure S7), further corroborating chemical records (Supplementary Figure S2) and suggesting that bottom seawater intrusion did not occur. Wellhead incubations enriched Gamma- and Epsilon-proteobacteria lineages not observed in the downhole incubations (Figures 5A,B and Supplementary Figure S7A). These Gammaproteobacteria OTUs were related to sequences from ODP Hole 896A crustal fluids (Nigro et al., 2012). Epsilonproteobacteria were exclusive to wellhead deployments (Figure 5B). These sequences were classified to the genus *Arcobacter* and were highly similar to sequences from previous Hole U1301A wellhead enrichments (Baquiran et al., 2016).

Downhole incubations were dominated by lineages related to anaerobic thermophiles (Figures 5C,D and Supplementary Figures S7–S9). Firmicutes comprised between 6–87% of downhole samples (Figure 4A). Two high abundance OTUs, lineages within the class Clostridia, had high sequence identity (>99%) with a JdF observatory rust clone (Nakagawa et al., 2005) classified as *Ammonifex* sp. The Aminicenantes (OP8), reported in JdF crustal fluid communities (Huber et al., 2006; Jungbluth et al., 2013) and previous *in situ* mineral incubations (Orcutt et al., 2011a), were recovered from downhole incubated basalts (Figure 5D and Supplementary Figure S8A). Deltaproteobacteria predominantly clustered into 4 abundant lineages distantly related to cultured obligate thermophiles (Supplementary Figure S7B). Thermotogae, an anaerobic thermophilic phylum (Nesbo et al., 2015) reported previously in JdF crustal fluids (Jungbluth et al., 2016) and *in situ* enrichments (Orcutt et al., 2011a), was represented by two OTU lineages in downhole basalt incubations (Supplementary Figure S8B). Gammaproteobacteria, related to lineages from deep-sea and sediment environments, were recovered from downhole incubations (Supplementary Figure S7A). Other anaerobic thermophilic clades, some previously observed in substrate incubations and crustal fluids at this location (Orcutt et al., 2011a; Jungbluth et al., 2013, 2016), were detected in downhole deployments (Supplementary Figure S9).

### Functional Analyses of Crust-Attached Biofilm Versus Crustal Fluid Plankton

To assess possible functional differences between rock-attached biofilms versus the planktonic phase in the warm and anoxic JdF crustal subsurface, we compared our biofilm amplicon dataset to published JdF crustal fluid metagenomic libraries (Jungbluth et al., 2017a) through amplicon-based recruitment. A total of 79 metagenome assembled genomes (MAGs) with completeness levels ranging from 99 to 10% were reconstructed from the JdF crustal fluid metagenome assemblies (see Supplementary File 2 for stats of all MAGs). Twenty three of these MAGs contained Bacterial 16S rRNA genes that overlapped with the region amplified by our amplicon primers. Assemblies of 16S rRNA genes from MAGs were validated by read coverage across the length of each gene and in comparison to the contig in which it is encoded. Twelve of these 23 MAGs contained 16S rRNA genes with a at least 99% identity over at least 90% coverage of our biofilm 16S rRNA amplicon length (Supplementary Figures S10, S11). We interpret these 12 bins as “biofilm-linked”, representing planktonic taxa that have preferentially colonized FLOCS substrates (Figure 2). The remaining 11 reconstructed 16S rRNA genes do not meet our alignment threshold (i.e., they have substantially lower sequence identities to our biofilm amplicons; mean = 81.25%; maximum = 93.75%) and thus represent “planktonic” taxa that did not colonize FLOCS substrates. The phylogenetic affiliations of these bins, estimated by PhyloSift, and the closest NCBI matches to their 16S rRNA genes are reported in Supplementary Figure S10 and Supplementary File S1, respectively.

We did a similar comparison of our amplicon dataset to a metagenome that was recently published (Smith et al., 2019) from a biofilm formed on olivine in this same habitat. In our reassembly of this JdF olivine metagenome, we detected eight metagenome bins with 16S rRNA genes with matches to our biofilm OTUs (Supplementary Table S4). Of these eight, two of them (OTUs 50 and 120) were also detected in the JdF crustal fluid metagenome, and one of them (OTU 122) was detected in a metagenome from NP crustal fluids.

**FIGURE 6:**
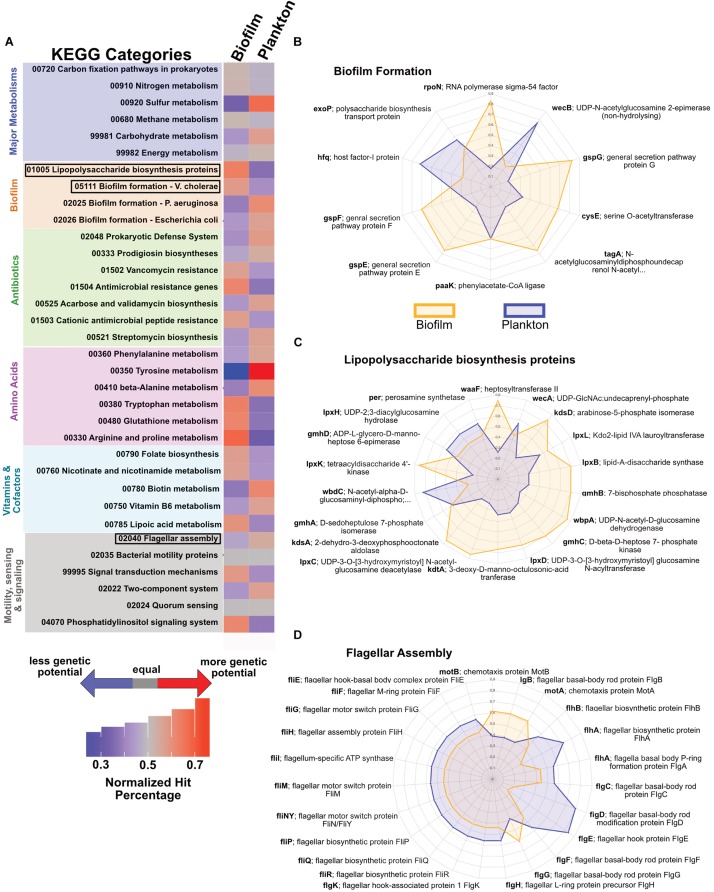
Potential functional differences between biofilm-linked and planktonic MAG fractions. **(A)** Relative abundance (per legend) of metagenomic reads binning into different KEGG categories between the biofilm-linked and plankton fractions, where gray indicates equal abundance in the two fractions, red indicates a higher percentage (i.e., more genetic potential in KEGG category), and blue indicates a lower percentage (i.e., less genetic potential). **(B–D)** Spider plots indicating the relative abundance (from 0 in the center to 1 on the outer edge) in the biofilm-linked (orange) and plankton (blue) fractions of particular genes in KEGG categories for biofilm formation **(B)**, lipopolysaccharide biosynthesis **(C)**, and flagellar assembly **(D)**.

Based on the partitioning of the 23 MAGs from JdF crustal fluid metagenomes into biofilm or planktonic categories, we examined the relative abundance of functional genes in each metagenome set (see Supplementary File S2 for the full analysis). For major metabolic pathways, there are only minor differences in the relative abundance for annotations related to carbon fixation, nitrogen cycling and energy metabolism. We observed a higher relative abundance of pathways associated with sulfur and carbohydrate metabolisms in the planktonic fraction (Figure 6A). The biofilm-linked MAGs had a higher relative abundance of genes related to biofilm formation and lipopolysaccharide biosynthesis (Figures 6B,C) and planktonic MAGs had a higher relative abundance of genes related to flagellar assembly (Figure 6D).

Hidden Markov Models were used to further evaluate the genetic potential of the JdF MAGs grouped into biofilm and plankton fractions (Table 1). Genetic markers associated with carbon fixation via the Wood-Ljungdahl pathway are detected in both fractions. Various sulfate and dissimilatory sulfite reductases (*i.e., dsrABCMK* and *dsrD*) were also observed in both categories, while a polysulfide-carrier heterodimer (*soxYZ*) associated with the *sox* thiosulfate oxidation pathway (Gregersen et al., 2011) is only detected in the biofilm fraction. Genes for three types of oxygen reductases (*ccoNOP*, *coxAB*, and *cydAB*) and dissimilatory nitrate reductases (*napA*, *narG*) are observed in both fractions, while dissimilatory nitrite reduction (*nirBD*) and denitrification (*nirS*) genes are only observed in the biofilm-linked MAGs. Five groups of hydrogenases (Groups 1, 3b, 3c 3d, 4) are also observed in both biofilm and plankton fractions. Genes associated with the dissimilarity iron reduction and oxidation were absent from the planktonic and biofilm fractions.

### Comparing Rock-Attached Biofilm Community Overlap With Crustal Fluid Metagenomes

**FIGURE 7:**
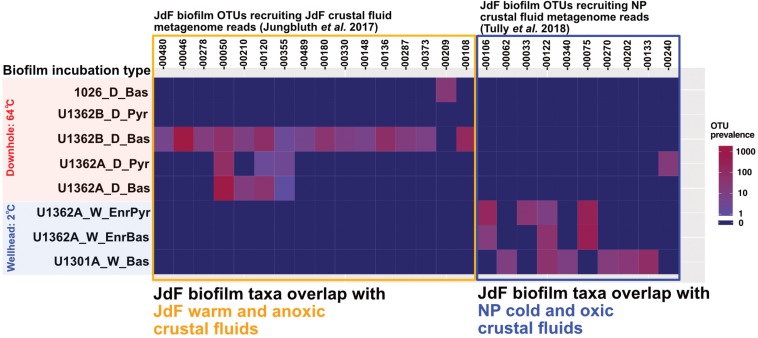
Heatmap depicting the prevalence of JdF biofilm OTUs recruiting metagenomic reads from crustal fluids [Juan de Fuca (JdF) and North Pond (NP)] across all incubation types and substrates in our experiments. Numbers across x-axis refer to JdF biofilm OTUs recruiting crustal fluid metagenome reads. The y-axis shows the prevalence of OTUs with crustal fluid metagenomic matches across all substrate types and incubation conditions in this study. OTUs recovered from JdF downhole deployments recruit metagenomic reads from the warm and anoxic JdF crustal fluid but not the cool and oxic NP crustal fluid. In contrast, OTUs from the JdF wellhead deployments recruit metagenomic reads from the cold and oxic NP crustal fluids almost exclusively.

Biofilm communities in the downhole FLOCS incubations (i.e., sourcing subsurface crustal fluids and incubated within the JdF warm and anoxic subsurface) recruit metagenomic 16S rRNA gene reads from the JdF crustal fluids (Jungbluth et al., 2017b) exclusively (Figure 7). By contrast, FLOCS deployed at CORK wellheads (*i.e.*, sourcing subsurface JdF crustal fluids but incubated under cold conditions at the observatory wellhead) recruit metagenomic 16S rRNA gene reads mostly from the cool and oxic NP crustal fluids (Tully et al., 2018).

## Discussion

Both rock-attached biofilms and free-floating plankton live in the crustal subseafloor; therefore, assessing the factors governing plankton recruitment onto crustal surfaces and, subsequently, differences in composition and potential activities of biofilm and plankton is necessary for a comprehensive ecological description of this habitat. In this study, we examine the influence of incubation conditions on subseafloor rock-attached biofilm composition (Figure 1). Further, we examine the partitioning of microbial groups into biofilms from the planktonic phase. Our results indicate that this process is influenced by environmental conditions (Figures 4, 5, 7), resulting in potential functional differences between these crustal community types (Figure 6).

### Biofilm Development on Mineral Surfaces Deployed in Crustal Fluids for 1–6 Years

Biofilms formed on the substrate colonization experiments to a cell density of up to 10^3^–10^6^ cells per gram of rock based on quantitative PCR analysis of 16S rRNA genes in pooled DNA extracts (Supplementary Table S3). Some samples had DNA concentrations that were below quantification limits. Thus, the biofilms had relatively low biomass, which is expected for a subsurface ecosystem (Orcutt et al., 2011a; Smith et al., 2011, 2016), as compared to 10^7^–10^9^ gene copies per gram of seafloor-exposed basalt (Santelli et al., 2008).

We confirmed substrate colonization with SEM and observed various cell and mineral precipitate morphologies (Figure 3). In contrast to earlier experiments at this location (Orcutt et al., 2011a), twisted iron oxyhydroxide stalk morphotypes (previously attributed to iron oxidizing bacteria) were not observed. In the previous study, intrusion of oxygen-rich bottom seawater into the subsurface environment, due to leaky borehole conditions, was hypothesized to favor microaerophilic iron oxidation. Temporal records of dissolved ions do not indicate oxygen intrusion in these new experiments (Supplementary Figure S2), explaining the absence of the twisted-stalk morphotype. The origin of the morphotypes in the current experiments cannot be explained by this dataset alone.

### Assessing Possible Sequence Contamination in Low Biomass Subsurface Samples

Low biomass in this environment is a common issue (Salter et al., 2014; Morono and Inagaki, 2016; Labonte et al., 2017; Sheik et al., 2018); thus, our 16S RNA gene sequence datasets needed to be rigorously evaluated for possible sequence contamination. Having sequenced multiple DNA extraction blanks, we were able to screen our libraries for spurious sequences following the philosophy espoused in a recent study of other low biomass subsurface samples (Sheik et al., 2018). This led to the development of a new analysis pipeline – *TaxonSluice* – to enable automated and reproducible screening of amplicon datasets with corresponding sequence blanks. Applying the *TaxonSluice* algorithm to our dataset culled 30.4% percent of high-quality sequences from incubation samples as potential contaminants (Supplementary Table S3) which are excluded in the ecological analysis below.

### Environmental Factors Influencing Biofilm Development in the Crustal Subsurface

JdF crustal fluid taxa composition exhibits variability (Jungbluth et al., 2013; Tully et al., 2018). Despite this variability, the microbial clades comprising downhole biofilm are related to anaerobic thermophilic groups previously observed in crustal fluids at this site; *e.g.*, Firmicutes, Deltaproteobacteria, Aminicenantes/OP8, and Thermotogae (Figures 4, 5, and Supplementary Figures S7–S9). In contrast, the biofilms seeded by the same crustal fluid but incubated in cold (2°C) conditions were comprised of psychrophilic Gamma- and Epsilon-proteobacteria as reported before (Baquiran et al., 2016). These groups are also common in other environments, where crustal rocks are exposed to bottom seawater (Sylvan et al., 2013). Remarkably, there is also overlap between our JdF wellhead incubation communities and taxa in cool and oxic crustal fluids from NP on the Mid-Atlantic Ridge (Figure 7). These observations indicate that temperature, and possibly oxygen, is a major environmental factor driving rock-attached biofilm community assembly in crustal ecosystems. This ecosystem structuring driver has also been proposed at a global level (Edwards et al., 2012).

Other possible ecological drivers of rock-attached biofilm community structure are less clear. The warmer downhole incubations have higher species richness compared to the cooler downhole incubations (Figure 4B), suggesting reduced niche space in the cooler conditions. Downhole incubations lasted several years longer than the cooler incubations (Supplementary Table S2), so time may play a role in the diversification of the warmer biofilms, as suggested elsewhere (Santelli et al., 2008, 2009). Ordination patterns are not driven by rock substrate type (*i.e.*, basalts versus metal sulfides; Figure 4C), regardless of incubation location. This is consistent with earlier analyses of similar subseafloor mineral incubations (Orcutt et al., 2011a) but different than prior indications that mineralogy structures rock-attached microbial communities (Toner et al., 2013; Smith et al., 2016). Thus, the role of mineralogy on crustal ecosystem development is unclear and requires further investigation.

### Common Microbial Groups Across Crustal Subsurface Ecosystems

Similarities between wellhead biofilm members and taxa in crustal fluids from a different cool and oxic crustal subsurface ecosystem (Figures 5, 7) suggests that some lineages (e.g., *Colwellia* and *Arcobacter*) represent globally distributed denizens of the crustal subsurface biosphere. Although rare in the anoxic warm JdF crustal fluids, these cold-adapted aerobic groups are present in the aquifer and respond to changing environmental conditions (e.g., temperature) to dominate wellhead incubations (Figure 4A and Supplementary Figure S12). The consistency of biofilm community structure developing under cool wellhead conditions [i.e., the similarity of the *Colwellia* and *Arcobacter* spp. between this study and that of Baquiran et al. (2016)], and observations of these groups at this site previously (Huber et al., 2006; Jungbluth et al., 2013), suggests that some groups are consistently rare and responsive in the highly geochemically and thermally altered JdF crustal fluids. A taxonomic inventory of rocks from the cool and oxic subsurface at NP (Jorgensen and Zhao, 2016) also indicates an enrichment in Alteromonadales Gammaproteobacteria (the family in which *Colwellia* spp. reside) on subsurface basalts. We hypothesize that these microbial groups are sourced from bottom seawater and persist even in highly altered crustal fluids to rebound when conditions become favorable again.

Within the Firmicutes group, we note that the marine *Ca.* Desulfopertinax/terrestrial *Ca.* Desulforudis lineage, commonly observed in JdF crustal fluids and terrestrial subsurface sites (Jungbluth et al., 2017b), was not detected in our incubations (Figure 5C). Genetic repertoires of these closely related candidate taxa suggest the potential for flagellar-based motility. Glycosyltransferases and diphosphate-sugar epimerases, genes involved in biofilm formation, are detected in the genome of the marine *Ca.* Desulfopertinax cowenii and are absent in its terrestrial subsurface counterpart *Ca.* Desulforudis audaxviator. Our observations indicate that this lineage, despite having the genetic potential for biofilm colonization, may favor a planktonic rather than rock-attached lifestyle in our incubation conditions. However, the recent metagenomic analysis of a biofilm formed on olivine in the JdF crustal subsurface did detect this group (Smith et al., 2019), perhaps indicating that their presence in biofilms is dictated by specific mineral types.

### Variable Functional Potential Between Rock-Attached and Planktonic Crustal Communities

Our partitioning of crustal fluid metagenomes into “biofilm-linked” and “planktonic” categories (Figure 2), suggests some functional differences between these compositionally distinct yet inter-related crustal communities (Figure 6). While this is an imperfect comparison, the observation that genes related to biofilm formation are more abundant in the biofilm fraction, while the abundance of motility-related genes is higher in the planktonic fraction, lends confidence to our approach. Also, our biofilm amplicon sequences matched 16S rRNA genes from 16S rRNA gene-bearing MAGs from a biofilm formed on olivine in the JdF crustal subsurface (Supplementary Table S4), with overlap with the groups identified from the JdF crustal metagenome (Figure 7), corroborating our interpretation of the biofilm lifestyle of these groups. It should be noted, however, that the functional potential encoded in MAGs lacking 16S rRNA genes, and missing genetic information from incomplete MAGs is not considered here, so this is a qualitative assessment.

Following this approach, we observe genes for multiple carbon fixation pathways (Wood-Ljungdahl, 3HP, 3HP/4HB) present in both biofilm and planktonic fractions in similar relative abundances, with the Calvin cycle gene RuBisCO only observed in plankton (Table 1). Prior studies have documented carbon fixation potential in cool and oxic subsurface crustal fluids (Meyer et al., 2016), in seafloor-exposed basalt biofilms (Orcutt et al., 2015) and in the anoxic subsurface of the JdF (Carr et al., 2019; Smith et al., 2019). Stable isotope assays suggest that carbon fixation in JdF crustal fluids can occur (McCarthy et al., 2010) although the system is net heterotrophic with a loss of organic matter (Lang et al., 2006; Lin et al., 2012). Our results support the potential for carbon fixation, performed by both biofilm and plankton communities, in the JdF subsurface crust.

By contrast, genes involved in sulfur cycling, despite being present in both fractions, may be relatively enriched in the plankton fraction (Figure 6 and Table 1). Sulfate is the dominant oxidant in this system (Wheat et al., 2010), thus sulfate reduction would be expected as a dominant process if appropriate electron donors were available. Microbial sulfate reduction and microbial groups associated with this process have been detected in JdF crustal fluids (Robador et al., 2014); however, *in situ* rates of this process are unknown. The potential for microbial sulfate reduction on the rocks has also been documented by sulfur stable isotopes (Lever et al., 2013), and recent metagenomic analysis of a biofilm formed on olivine in the JdF subsurface detected sulfate reduction capability in several MAGs (Smith et al., 2019).

Determining the genetic potential for iron cycling processes in this environment is challenging due to the dearth of fully characterized iron cycling pathways (He et al., 2017). The recent metagenomic study of a biofilm on olivine did not detect the *cyc2* genes (Smith et al., 2019). These genes are thought to be indicative of neutrophilic, microaerobic iron oxidation (Barco et al., 2015; Chan et al., 2018); which would be consistent with their absence from the anaerobic JdF crustal system. We detect *cyc2* in the JdF crustal fluid metagenome (data not shown); however, the subset of MAGs used in our analyses did not contain this gene. Iron is relatively abundant in the host rocks, as iron oxides comprise roughly 10% of the basalt (by weight) in this system, and iron oxyhydroxides are the second most abundant mineral alteration product. These phases indicate the potential for iron oxidation/reduction. Thermodynamic calculations suggest that utilization of iron is energetically favorable (Boettger et al., 2013). Theoretical calculations suggest that iron oxidation could potentially support chemolithoautotrophy in basalt ecosystems (Bach and Edwards, 2003), but further work is needed to fully characterize iron cycling processes in the crustal ecosystem.

The possibility of nitrate/nitrite oxidation/reduction in the Juan de Fuca crustal subsurface is controversial. Nitrate concentrations in crustal fluids from this environment are extremely low, near limits of detection [high nM to low μM; (Lin et al., 2012)] and are argued to be artifacts of drilling (Wheat et al., 2010) as nitrate is quickly lost from recharging fluids that enter this crustal outcrop system (Wheat et al., 2013). Despite this, Nitrospirae (nitrate oxidizers) are commonly detected in this crustal ecosystem (Jungbluth et al., 2017a). Here, we observe genes involved in denitrification in the biofilm-linked MAGs (Table 1), and a recent metagenomic study of a JdF olivine biofilm also detects nitrate reduction pathways genes (Smith et al., 2019) suggesting that cryptic nitrogen cycling at this site may be a possible metabolic feature, although it is unclear if this preferentially occurs in crust-attached biofilms.

The reductant-oxidant pairs in the JdF subsurface that fuel chemotrophy are unclear. Hydrogen and methane in JdF fluids could provide very low levels of free energy when coupled to various electron acceptors (Lin et al., 2012, 2014; Boettger et al., 2013; Robador et al., 2016). We detect Group 1 hydrogenases (hydrogen oxidation) typical of hydrogen consuming microorganisms (Peters et al., 2015) in the biofilm-linked metagenomic fraction. We did not detect methane cycling genes; however, 16S rRNA gene-containing MAGs used in this study only targeted Bacteria; thus, putative archaeal methanotrophs were not included in the analysis. Interestingly, uncultivated Archaea containing genes associated with methyl cycling (*mtrH*) but lacking *mrcA* have been recently reported from JdF crustal fluids (Carr et al., 2019). We detected genes coding for carbon monoxide dehydrogenase (Table 1). Furthermore, we observe genes associated with carbon monoxide oxidation in the biofilm-linked metagenomic fraction, expanding the potential for carboxydotrophy, recently reported for uncultivated Archaea in this system (Carr et al., 2019), to the Domain Bacteria. Carbon monoxide is known as an energy rich reductant in other subsurface ecosystems (Canovas et al., 2017).

The use of amino acids (Figure 6A) is supported by the consistent observation of the uncultivated Aminicenantes/OP8 group at this site (Orcutt et al., 2011a; Jungbluth et al., 2013, 2016; Smith et al., 2016). This group, also observed in terrestrial deep biosphere sites (Farag et al., 2014), is suggested to be involved in amino acid metabolism and peptide scavenging from cadaverous cells (Rinke et al., 2013; Sharon et al., 2015; Robbins et al., 2016). Amino acid and carbohydrate utilization potential is also detected in an olivine biofilm incubated at this site (Smith et al., 2019). Thus, despite the relatively minor contribution of necromass as a component of overall energy in deep marine sedimentary ecosystems (Bradley et al., 2018), its assimilation may support heterotrophy in marine and terrestrial crustal deep biosphere environments.

## Conclusion

The nature of biofilm cellular recruitment from plankton in subseafloor crust is explored in this study. We show that temperature, and possibly redox state, drive recruitment of planktonic cells from crustal fluids into mineral-attached biofilms, supporting prior hypotheses about the global ecological structuring of this habitat (Edwards et al., 2012). Comparisons of biofilm communities from this study against crustal fluid communities from JdF and NP reveal global crustal biosphere connectivity by identifying related microbial groups that persist through changing environmental conditions in different subseafloor crustal aquifers. Further, despite many similarities, emergent genetic differences between rock-attached biofilm and planktonic communities inhabiting subseafloor crust are highlighted. Overall, we address a major gap in deep biosphere ecology by reporting the community drivers and genetic potential for geomicrobiological activity localized to biofilms attached to subseafloor crust, the largest contiguous habitat on Earth. As estimates of global biomass in ocean crust are beginning to emerge (Heberling et al., 2010; Bar-On et al., 2018), we emphasize that biomass partitioning into plankton and biofilm in this habitat, each community type existing at a different cellular density, is necessary for overall estimate robustness and proper ecological interpretation.

## Data Availability

The datasets generated for this study can be found in NCBI Short Read Archive, BioProject PRJNA472057; BioSample accession numbers: SAMN09228041–SAMN09228055.

## Author Contributions

BO and CW conceived the study. GR, BO, and CW conducted the field deployments/recoveries. GR, BO, CW, AL, and TD performed the sample analyses. GR and AG performed the bioinformatic analyses and developed the software tools. GR, AG, CW, and BO interpreted the data. GR wrote the manuscript with input from all authors.

## Conflict of Interest Statement

The authors declare that the research was conducted in the absence of any commercial or financial relationships that could be construed as a potential conflict of interest.

## Footnotes

1 https://github.com/Arkadiy-Garber/taxonsluice
