# Supplementary material for: Ecology of Subseafloor Crustal Biofilms
Source: Front Microbiol. 2019 Aug 28;10:1983. doi: 10.3389/fmicb.2019.01983 (PMC6736579; doi:10.3389/fmicb.2019.01983)
Supplement: Supplementary file 3 [file Data_Sheet_3.PDF]

Frontiers in Microbiology Journal, Extreme Microbiology Section
Supplemental Information

Title: Ecology of Subseafloor Crustal Biofilms

Authors: Gustavo A. Ramírez[1], Arkadiy I. Garber[2], Aurélien Lecoeuvre[3,4], Timothy D'Angelo[3], C. Geoffrey Wheat[5] and Beth N. Orcutt[3*]

Author affiliations:
1: University of Rhode Island, Graduate School of Oceanography, Narragansett, RI, USA.
2: University of Montana, Division of Biological Sciences, Missoula, MT, USA.
3: Bigelow Laboratory for Ocean Sciences, East Boothbay, ME, USA.
4: University of West Brittany, France.
5: University of Alaska Fairbanks, Fairbanks, AK, USA.

*: Corresponding author: Dr. Beth N. Orcutt; Bigelow Laboratory for Ocean Sciences; 60 Bigelow Drive, PO Box 380; East Boothbay, ME, 04544, USA; +01 (207) 315-2567; borcutt@bigelow.org

Keywords: deep biosphere, subseafloor, oceanic crust, Juan de Fuca, CORK, FLOCS, low biomass

Supplemental Materials and Methods

FLOCS deployment configuration and recovery

Minerals used in the experiments were autoclaved prior to assembly of the FLOCS, after the FLOCS components had been thoroughly cleaned via acid-washing. Following FLOCS assembly, the FLOCS contents including substrates were flushed with ethanol and ultrapure deionized water followed by a second flush with commercially available sterile filtered water (Sigma-Aldrich product number S9148). In addition to crushed basalt and pyrite substrates (used for DNA extraction), polished autoclaved chips of various substrates were mounted on plastic grids in the FLOCS units for subsequent electron microscopy analyses. Borehole fluids were pumped into FLOCS chambers by OsmoSampler pumps (1, 2) at an intake rate of ~1 ml d^{-1}. "Enrichment" experiments (Table 1) include a second pump that injected a solution into the intake of the FLOCS resulting in slightly elevated phosphate and nitrate concentrations of 8 μM (3, 4). FLOCS were recovered and processed following established protocols (5, 6). Samples for DNA analyses were stored at –80°C for shore-based analyses. Samples for scanning electron microscopy where fixed shipboard in 4% paraformaldehyde in sterile filtered 1xPBS buffer, then rinsed and stored at –20ºC in a 1:1 mixture of 1xPBS and ethanol.

Electron microscopy analyses

The ethanol-dehydrated polished chips were mounted on Al stubs with adhesive discs and conductive Al/Ni tape (Electron Microscopy Sciences parts 77825-12 and 77813), sputter coated with gold, and analyzed with a Zeiss Supra25 SEM at the Bigelow Laboratory for Ocean Sciences (operated with a 15-kV accelerating voltage). Samples from the other locations unfortunately were lost during transition between laboratories and could not be analyzed. Because the samples were not critical point dried prior to sputtering, it is likely that cell integrity was compromised, and thus, the SEM analysis was primarily useful for looking at

secondary mineral precipitates, which can have characteristic shapes in this environment (see Orcutt et al. 2011). While the Hole U1362B downhole DNA samples represent pooled DNA extracts from multiple rock chips, the SEM analyses could be separated by individual chip, allowing investigation of colonization patterns on multiple different basalts and sulfides, as well as other rock types that were not investigated for DNA analysis (i.e., iron oxides).

Geochemical Analysis

A time-stamp dissolved fluid chemistry record for each deployment set was collected by sectioning the Teflon tubing collection coil, as described elsewhere (7). Briefly, the effluent of shipboard-sectioned 1m Teflon tubing intervals is collected followed by subsample acidification ascribing to established protocols (3). Back on shore, samples undergo major and minor ion measurements using inductively coupled plasma mass spectroscopy (ICP-MS) as previously detailed (7).

DNA extraction

Approximately 6g of each substrate underwent total DNA extraction using the MOBIO PowerSoil® DNA Isolation Kit (MOBIO Laboratories, Inc.) with modification from manufacturer instructions to include a phenol:chloroform:isoamyl alcohol (24:25:1) extraction step after the cell lysis step (i.e. after addition of Solution C1 and incubation at 65ºC). Each batch of samples included procedural blanks. Replicate extracts were pooled and concentrated to 100 µl with vacuum centrifugation (SavantTM DNA SpeedVacTM Concentrator). Concentrated extracts were cleaned with the CleanAll DNA/RNA Clean Up and Concentration Kit (Norgen Biotek Corp, Thorold, Canada) and eluted in 20-50 µl of PCR-grade water. DNA concentration was measured with the Qubit™ dsDNA High Sensitivity Assay kit (InvitrogenTM, Molecular Probes®) and the Qubit™ 2.0 Fluorometer following the manufacturer protocols (detection limit 0.5 ng DNA μl^{-1}). Note: Other FLOCS incubated

substrates underwent total DNA extraction as described here but did not yield quantifiable/amplifiable extract quantities.

Quantitative PCR

To assess the abundance of bacteria in the samples, the bacterial 16S ribosomal (rRNA) gene was amplified with the PerfeCta SYBR Fast Mix for iQ5 system Kit (Quanta Biosciences) with the *Bac8Fmod* forward primer (8) and *Bac338Rabc* reverse primer (9). Real time amplification was conducted with a BioRad™ iQ5 Optical system with the following conditions: an initial denaturation at 95°C for 1min; 40 cycles of denaturation (5s at 95°C), annealing (15s at 55°C) and elongation with measure of fluorescence (15s at 68°C); and a final melt curve from 55°C to 95°C. A linearized plasmid containing a nearly full-length bacterial 16S rRNA gene at known quantities was used as a copy number standard.

16S rRNA gene sequencing and data processing

Sequencing of the V1-V3 region of the 16S rRNA gene was performed by Research and Testing Laboratory (Lubbock, TX, USA) using the forward primer 28F (5′- GAG TTT GAT CNT GGC TCA G -3′) and reverse primer 388R (5’- TGC TGC CTC CCG TAG GAG T -3’) for Bacteria using 2 x 300 bp kits on the Illumina MiSeq platform. Sequences were processed with *mothur* v.1.34.4 (Schloss et al. 2009) following the *mothur* Illumina MiSeq Standard Operating Procedure (10). Paired reads were merged and all sequences with homopolymers longer than 8 base pairs were discarded. Merged reads were aligned to the *mothur*-recreated Silva SEED v119 database (11) and subsequently pre-clustered at 1% dissimilarity using the *pre.cluster* (diffs=2) command. Thus, generation of spurious sequences was mitigated by abundance ranking sequences and merging with rare sequences if sequences differed by 2 base pairs, as outlined elsewhere (Kozich et al. 2013). Chimera screening and removal from further downstream analyses was performed by implementation of *de novo* mode of UCHIME (12). A

distance matrix was generated for sequences ascribing to these parameters with subsequent clustering into Operational Taxonomical Units (OTUs) at 3% or less sequence dissimilarity using the average neighbor method. Taxonomic classification of OTUs were performed with *mothur* using the SILVA v119 database (13).

TaxonSluice

To identify potential contaminants from DNA extraction kits, we developed *TaxonSluice*. This tool, mock-community datasets examples and full documentation is publicly available (https://github.com/Arkadiy-Garber/taxonsluice). Briefly, *TaxonSluice* parses through i) sample-specific and ii) non-sample-specific blanks assessing, based on a user-defined (10% default, used in this study) abundance threshold, retention or "flagging" of each OTU (minimum abundance = 5) as a potential contaminant. If independent blanks were available for each sample extraction batch (as was the case with this study), *TaxonSluice* examines if OTUs absent from sample-specific blanks were present in other, non-sample-specific, blanks (Figure S1). This step further validates or negates OTUs present in their sample-specific blank in lower than threshold abundance as suspected contaminants and accounts for laboratory equipment and technique as additional contaminant sources.

Sequence analyses

OTU rarefaction and ordination analyses and visualizations were performed in *RStudio* version 0.98.1091 (14) using the *vegan* version 2.3-0 (15) and *phyloseq* (16) packages. Principle Coordinate Analysis (PCoA) was performed on Jaccard and Bray-Curtis dissimilarity matrixes calculated from a normalized (subsampled to n= 4,785 per sample) dataset with the *vegsdist()* command. Major clustering patterns were in agreement for both Jaccard and Bray-Curtis outcomes, the former takes into account abundance while the latter considers the sum of lesser values for a shared taxonomic unit between samples relative to the total numbers of species,

the Jaccard result is shown in Figure 3C. Additionally, to test the effect of normalization strategy on exploratory ordinations, we compared non-normalized, rarefied and, variance stabilized transformation [VST, recommended for sparse datasets] ordination results. All ordinations, show sample partitioning as a function of incubation temperature rather than substrate type in our dataset (Figure S13).

Phylogenetic analyses

Sequence alignments were performed using the high speed multiple sequence alignment program MAFFT with the command: mafft --maxiterate 1000 –localpair seqs.fasta > aligned.seqs.fasta. Maximum likelihood trees with 100 bootstrap support were constructed using the RAxML program using the following parameters: raxmlHPC -f a -m GTRGAMMA -p 12345 -x 12345 -# 100 -s aligned.seqs.fasta -n T.tree, -T 4 ML search + bootstrapping. Newick trees files were uploaded to FigTree v1.4.2 for visualization. The outgroup for all of our Bacterial trees was the Archaeon *Methanoculleus* spp. (Silva accession number: KJ004582).

Metagenomic analyses

Publically available metagenomes were downloaded from the NCBI Sequence Read Archive: JdF (NCBI BioSamples SAMNO3166137 and SAMO3166138 for Hole U1362A and U1362B fluids, respectively) and NP (NCBI BioSamples SAMN07571231-SAMN07571245 for Holes U1382A and U1383C at various sampling times and depths, see (17)). Metagenome reads were quality-trimmed using *trimmomatic* v.0.36 (18). High-quality paired-reads were then combined using *FLASH* v.1.2.11 (19). Paired and unpaired reads from each BioSample were assembled separately using *SPAdes* v.3.11.1 with the '–-meta' flag (20); the assembled scaffolds from the Juan de Fuca metagenome were merged using *Minimus2* (AMOS) v.1.3.0 (-D MINID = 98 -D

OVERLAP = 80) (21). Sequence alignment maps (SAMs) were generated using high-quality raw reads recruited to merged scaffolds using *Bowtie2* v.2.3.3.1 (22). SAMs were then used to extract coverage information using *SAMtools* v.1.4.1 (23) and the *jgi_summarize_bam_contig_depths* script, available within the *MetaBAT* tool suite (24). Binning was performed with *MetaBAT* v.32.4 (settings: --verysensitive, --sensitive, --specific, --veryspecific, --superspecific), *CONCOCT* 0.4.1, and *MaxBin* v.2.2.2 resulting in a total of 389 bins. These were subsequently merged into a non-redundant set of bins using *DASTool* v.1.1.0 (--search_engine blast --score_threshold 0.2) (25), and manually-curated in *Anvi'o* v.3 (26). Bin quality was assessed using *CheckM* v.1.0.7 (27). Reads corresponding to the 16S rRNA gene were extracted using *SortMeRNA* v.2.1 (28); these putative rRNA reads were then compared against the 16S rRNA gene amplicons from FLOCS biofilms using *BLAST* v.2.6.0 with a 99% match over 90% of the amplicon length threshold. Results were processed using a custom Python script.

Genome evaluation and annotation

Taxonomic affiliations of the 16S-containing MAGs were estimated using *PhyloSift* v.1.0.1 (29). Gene annotations were done using the KEGG Ontology system; assembled scaffolds were uploaded to *GhostKoala* (default parameters; genus_prokaryotes + family_eukaryotes). The output was downloaded and analyzed using custom python scripts, as well as the scripts available within *GhostKoalaParser* (https://github.com/edgraham/GhostKoalaParser) and custom python scripts. We also used a custom library of hidden Markov models (HMMs) focused on chemolithoautotrophic metabolisms, allowing us to further explore the metabolic potential of selected bins. This library includes metabolic markers described and implemented elsewhere (30), in addition to HMMs designed specifically for this study.

16S identification and curation from metagenome

16S genes were identified within the final set of curated bins using *checkm ssufinder* v.1.0.7 (27). To exclude potentially chimeric 16S sequences, we identified candidate 16S reads from the high-quality raw reads using *SortMeRNA* v.2.0 (28), and mapped those to the *SPAdes*-assembled 16S genes; coverage was estimated using *SAMtools*. Sequences with coverage scores inconsistent across the length of the reconstructed 16S, or with the overall coverage of the scaffold from which the gene was identified, were excluded from downstream analyses. The resulting set of 16S sequences were aligned using *MUSCLE* v.3.8.1551 (31) to exclude information outside of the range of the primers used to generate FLOCS amplicons.

Supplemental Tables

Table S1. Characteristics of the CORK observatories used for the mineral colonization experiments.

CORK ID	Latitude (N)	Longitude (W)	Year CORK installed	Ref.
1026B	47.9601	-127.9033	1996/2004	(32)
U1301A	47.8081	-127.9813	2004	(32)
U1362A	47.7610	-127.7612	2010	(33)
U1362B	47.7583	-127.7622	2010	(33)

Table S2. Characteristics of the mineral colonization experiments recovered in 2014 and examined in this study. mbsf, meters below seafloor.

CORK	Package	Year deployed	Sample Substrates	Incubation temp. (°C)	Deployment depth (mbsf)	Fluid source depth (mbsf)
1026B	Downhole	2008	Basalt	64	280	>280
U1301A	Wellhead	2013	Basalt	2	0	270
U1362A	Wellhead Enrichment	2013	Basalt, Pyrite	2	0	310
U1362A	Downhole	2010	Basalt, Pyrite	64	445	>430
U1362B	Downhole	2010	Basalt, Pyrite	64	295	>295

Table S3. DNA extract concentration and estimation of cell density based on bacterial 16S rRNA gene qPCR. Abbreviations: bdl, below detection limit (0.5 ng DNA*ul^{-1}); n.d., not determined. QC Sequences are the sequences per samples after executing the mothur pipeline. Screened sequences are the number of sequences per sample remaining after running all OTUs through the heuristic culling algorithm implemented using *TaxonSluice*. Note: Each MOBIO Powersoil extraction contained 0.6-0.75g of substrate, approximately 10-15 replicate extractions were combined and concentrated before sequencing.

Sample	ID	g_{rock}	ng DNA g_{rock}^{-1}	16S rRNA genes ul^{-1}	QC Seqs.	*TaxonSluice* Seqs.
U1362A wellhead Enr. Basalt	U1362A-1	8.8	8.7	$1 \pm 0.4 \times 10^{6}$	35,148	33,033
U1362A wellhead Enr. Pyrite	U1362A-2	5.3	0.6	$8 \pm 0.4 \times 10^{2}$	35,494	34,275
U1301A wellhead Basalts	U1301-A	6.8	0.7	$2 \pm 3 \times 10^{4}$	33,881	31,484
U1362A downhole Basalts	150320-1	3.0	3.1	n.d.	31,021	26,257
U1362A downhole Pyrite	150320-2	4.6	1.8	n.d.	40,994	31,653
U1362B downhole Basalts	U1362B-1	6.5	bdl	$1 \pm 0.2 \times 10^{3}$	64,437	37,524
U1362B downhole Pyrite	U1362B-2	5.1	bdl	n.d.	13,261	4,785
1026B downhole basalts	1026B-4	6.5	bdl	n.d.	42,533	10,799

Table S4: Summary of BLAST hits (>99% identity, >90% amplicon length) between FLOCS amplicons and JdF olivine biofilm metagenome from Smith *et al*., 2019.

OTU	Match source	Most resolved OTU taxonomic affiliation	OTU status as a FLOCS member
Otu 1	Megahit contig	*Propionibacterium* sp.	Contaminant
Otu 20	Megahit and SPAdes contig	Enterobacteriaceae	Contaminant
Otu 50	Megahit and SPAdes contig	Candidate Division OP8	Environmental
Otu 74	SPAdes contig	*Lactobacillus* sp.	Environmental
Otu 120	JdFRolivine-4 from Smith *et al*., 2019	Candidate Division OP1	Environmental
Otu 122	Megahit and SPAdes contig	*Colwellia* sp.	Environmental
Otu 172	Megahit contig	*Thermosipho* sp.	Environmental
Otu 502	Megahit contig	*Halomonas* sp.	Environmental
Otu 656	JdFRolivine-3 from Smith *et al*., 2019	Candidate Division OP1	Environmental
Otu 3703	Megahit contig	*Propionibacterium* sp.	Discarded due to low abundance

CheckM ssu-finder identified SSU genes from three of the bins from Smith *et al*., 2019: JdFRolivine-3, JdFRolivine-4, and JdFRolivine-9. FLOCS amplicons were matched to two bins from Smith *et al*., 2019 (JdFRolivine-3 and JdFRolivine-4). We then performed our own assembly of the metagenomic dataset using Megahit and SPAdes and matched an additional 8 amplicons to the assembled contigs. Two of the amplicons that matched our assembled contigs were previously identified as contaminants using the *taxonsluice* algorithm, suggesting that some contaminant DNA sequences may have persisted through metagenomic assembly. However, we see no indication that potential contaminant sequences were binned into the final published Bacterial MAGs.

Supplemental Figures

Supplemental Figure S1. Algorithm implemented in *TaxonSluice* to identify OTUs. *TaxonSluice* may be downloaded here: https://github.com/Arkadiy-Garber/taxonsluice. See documentation for inputs and dependencies.

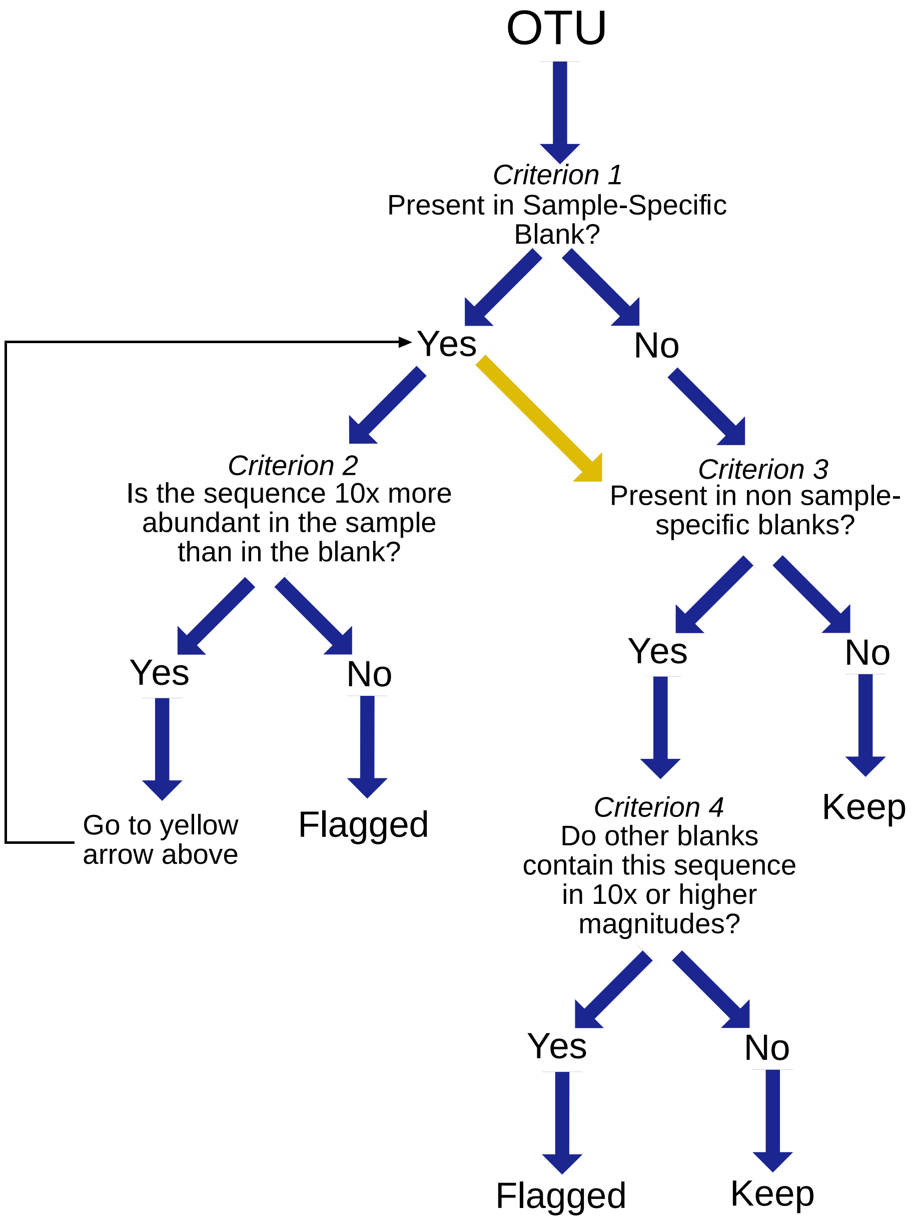

Supplemental Figure S2. A) Manganese and B) Sulfate ion concentrations plotted as of function of time. The dashed horizontal blue and gold lines are reported values for background seawater and JdF hydrothermal formation fluids at these sites, respectively.

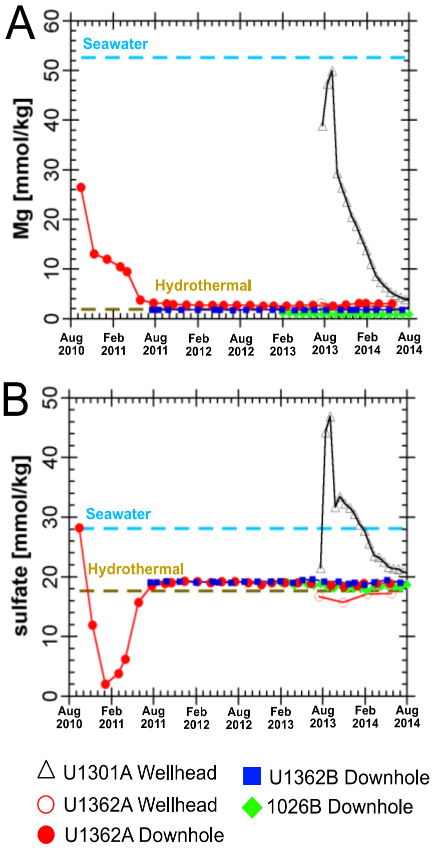

Supplemental Figure S3. Select scanning electron micrographs (SEM) from rock chips incubated at Hole U1362B on grid B196. See main text Figure 2 for legend. Scale bar in each are 10 μm.

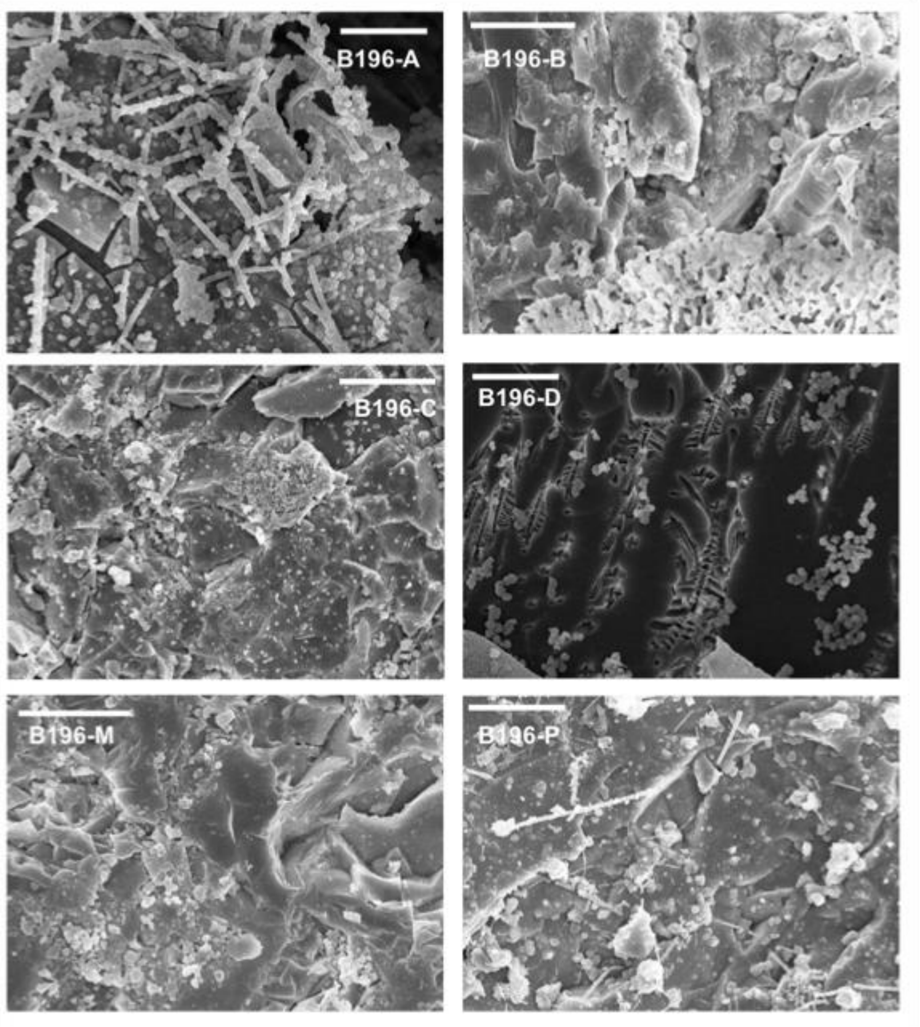

Supplemental Figure S4. Select scanning electron micrographs (SEM) from rock chips incubated at Hole U1362B on grid B197. See main text Figure 2 for legend. Scale bar in each are 10 μm.

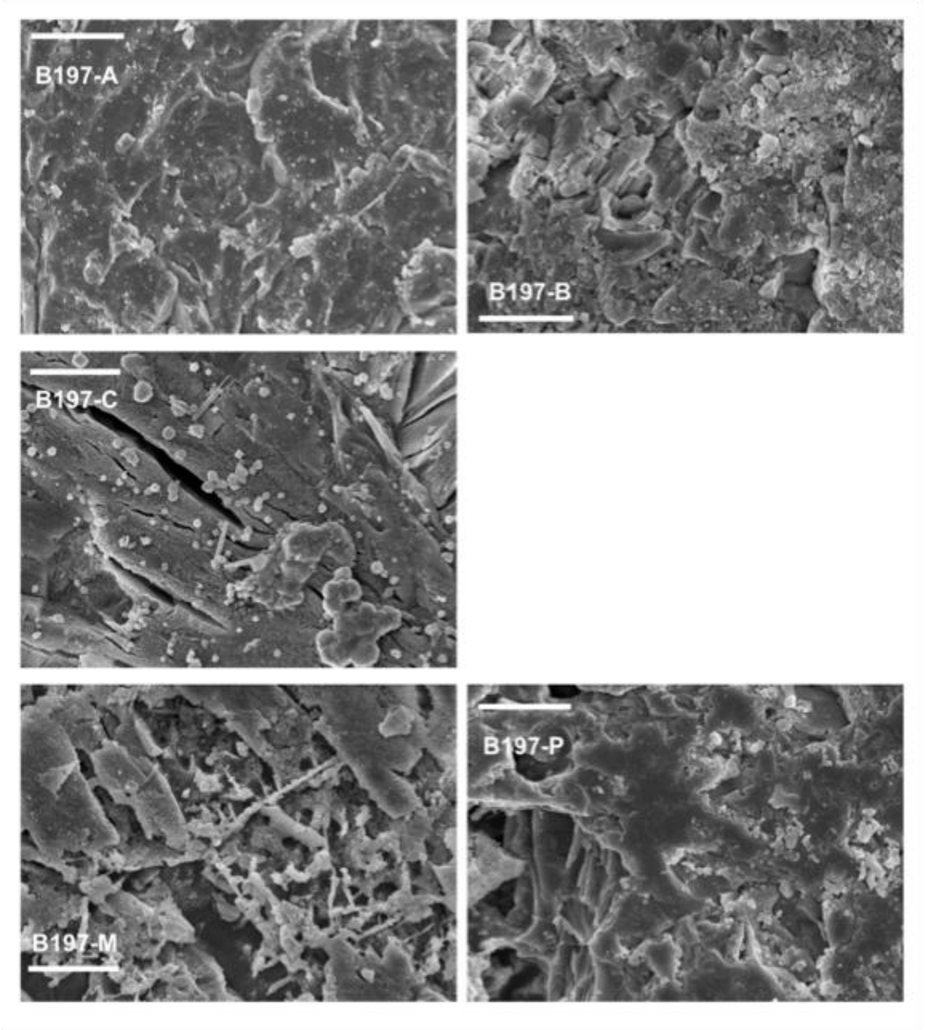

Supplemental Figure S5. Select scanning electron micrographs (SEM) from rock chips incubated at Hole U1362B on grid B199. See main text Figure 2 for legend. Scale bar in each are 10 μm.

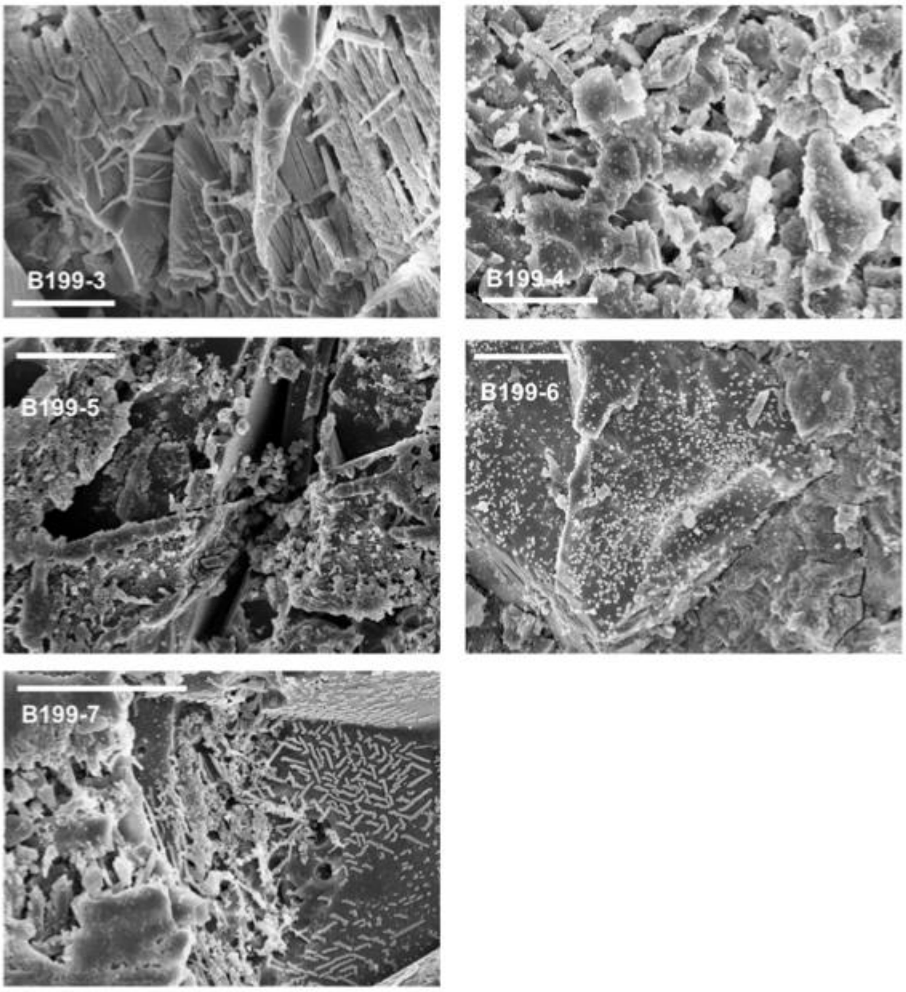

Supplemental Figure S6. Select scanning electron micrographs (SEM) from rock chips incubated at Hole U1362B on grid B201. See main text Figure 2 for legend. Scale bar in each are 10 μm.

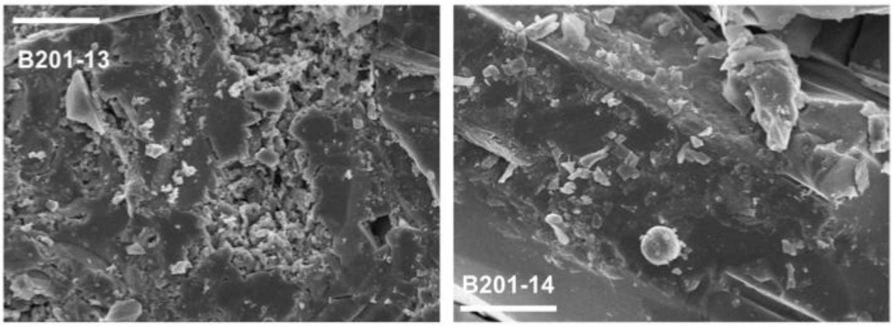

Supplemental Figure S7. A) Phylogenetic tree of Gammaproteobacteria from wellhead and downhole incubated FLOCS. The collapsed section of the tree is presented as Figure 3A in the main text. B) Phylogenetic tree of the Deltaproteobacteria recovered in this study. The Epsilonproteobacteria, collapsed in this figure, are shown as Figure 3B in the main text.

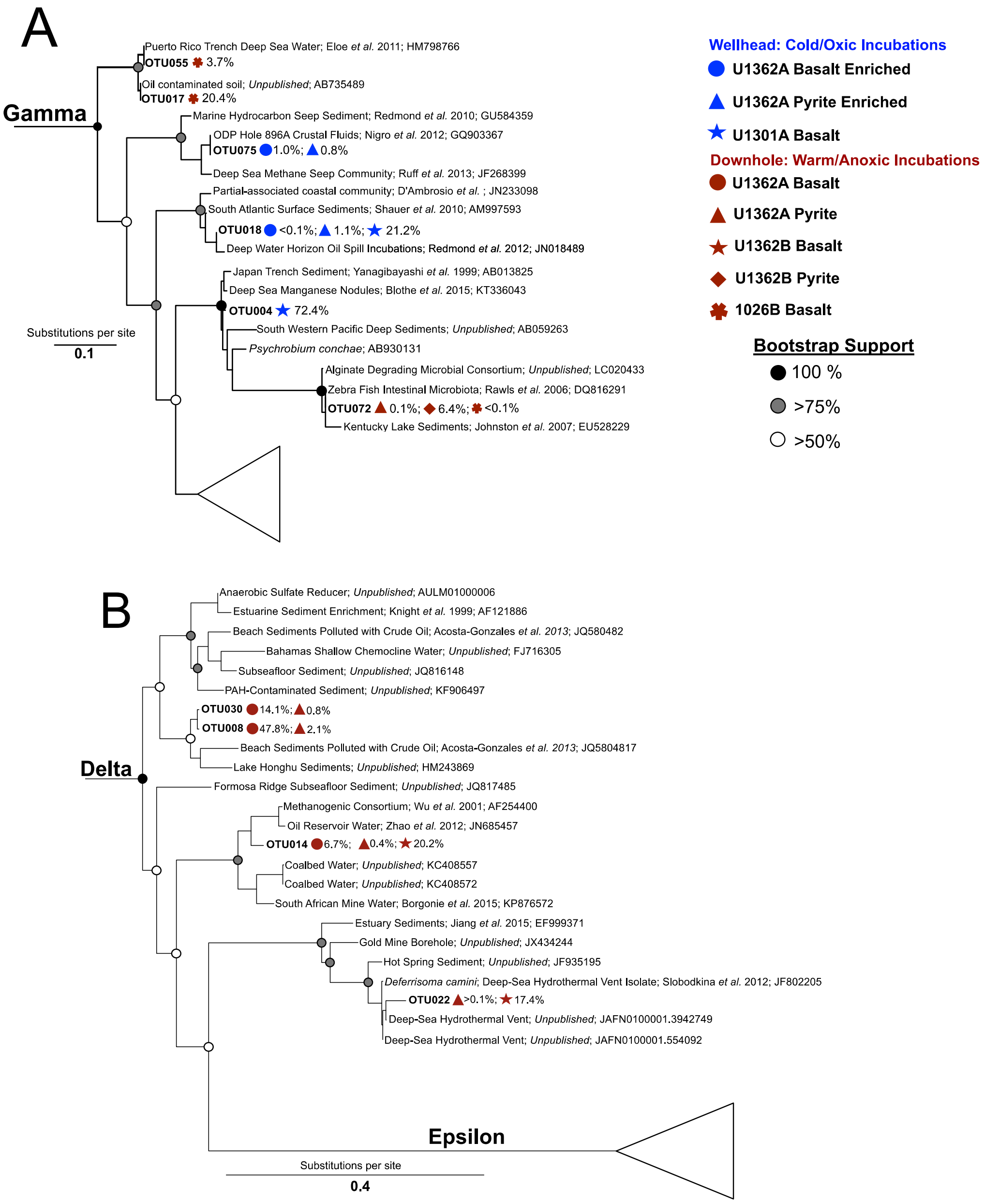

Supplemental Figure S8. A) Complete phylogenetic tree of high abundance Aminicenantes recovered from downhole incubated FLOCS. The collapsed section of the tree is presented as Figure 3D in the main text. B) Phylogenetic tree of high abundance Thermotogae phylum sequences recovered in this study.

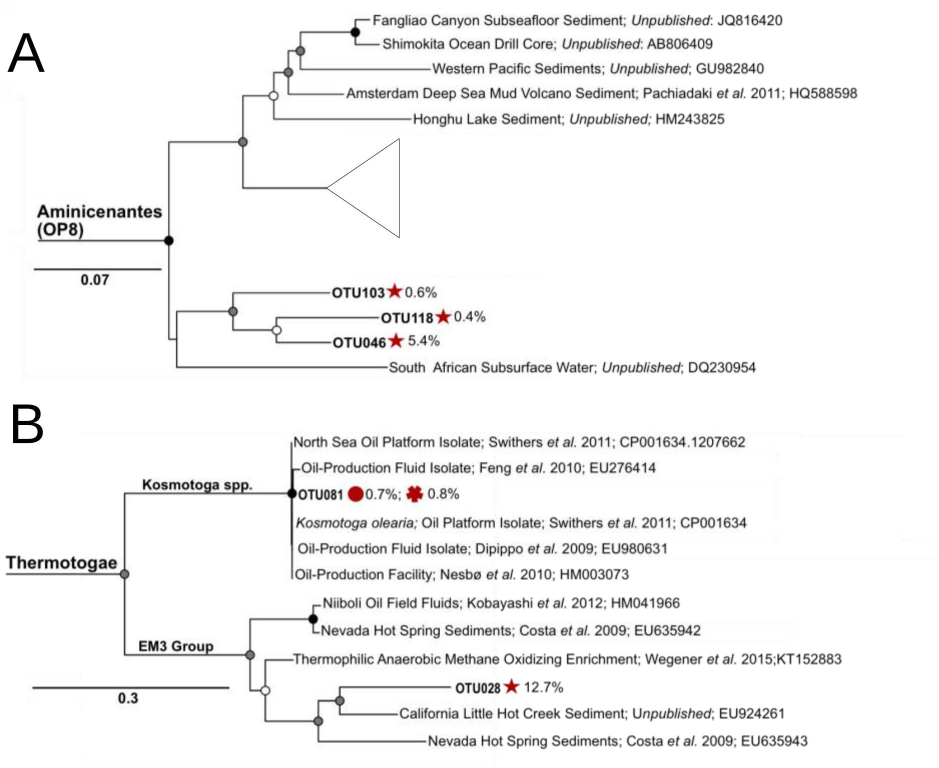

Supplemental Figure S9. A) Phylogenetic tree of the Actinobacteria, Spirochetes, Bacteroidetes phyla and Betaproteobacteria class for various downhole incubated deployments. B) Phylogenetic tree of OTUs recovered from downhole incubations assigned to the phylum Chloroflexi. C) Phylogenetic tree of OTUs recovered from downhole incubations assigned to the phylum Deferribacteres.

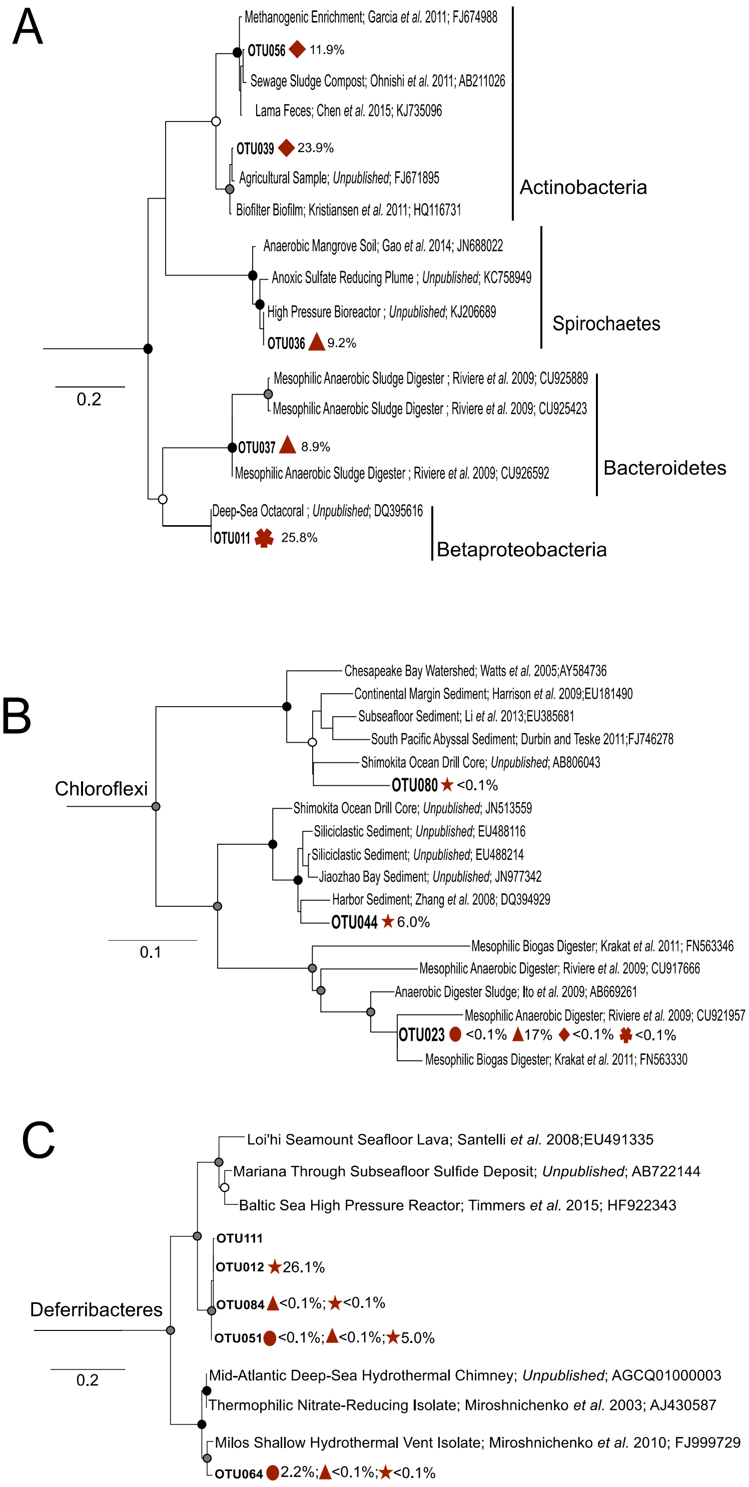

Supplemental Figure S10. Final set of 11 biofilm-linked and 12 plankton bins containing bacterial SSU rRNA genes with consistent coverage scores.

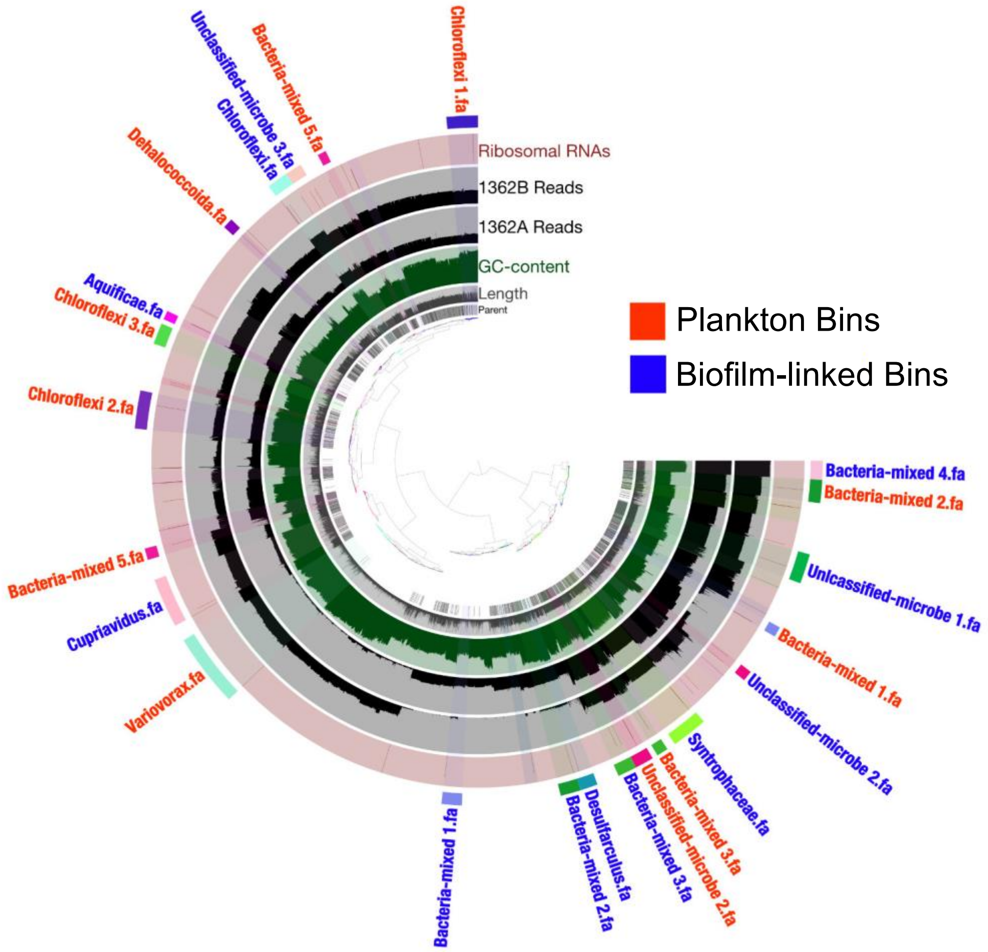

Supplemental Figure S11. Coverage estimation of high-quality candidate 16S rRNA gene reads identified with *CheckM ssufinder* mapped against *SPAdes*-assembled 16S rRNA genes using *SAMtools*.

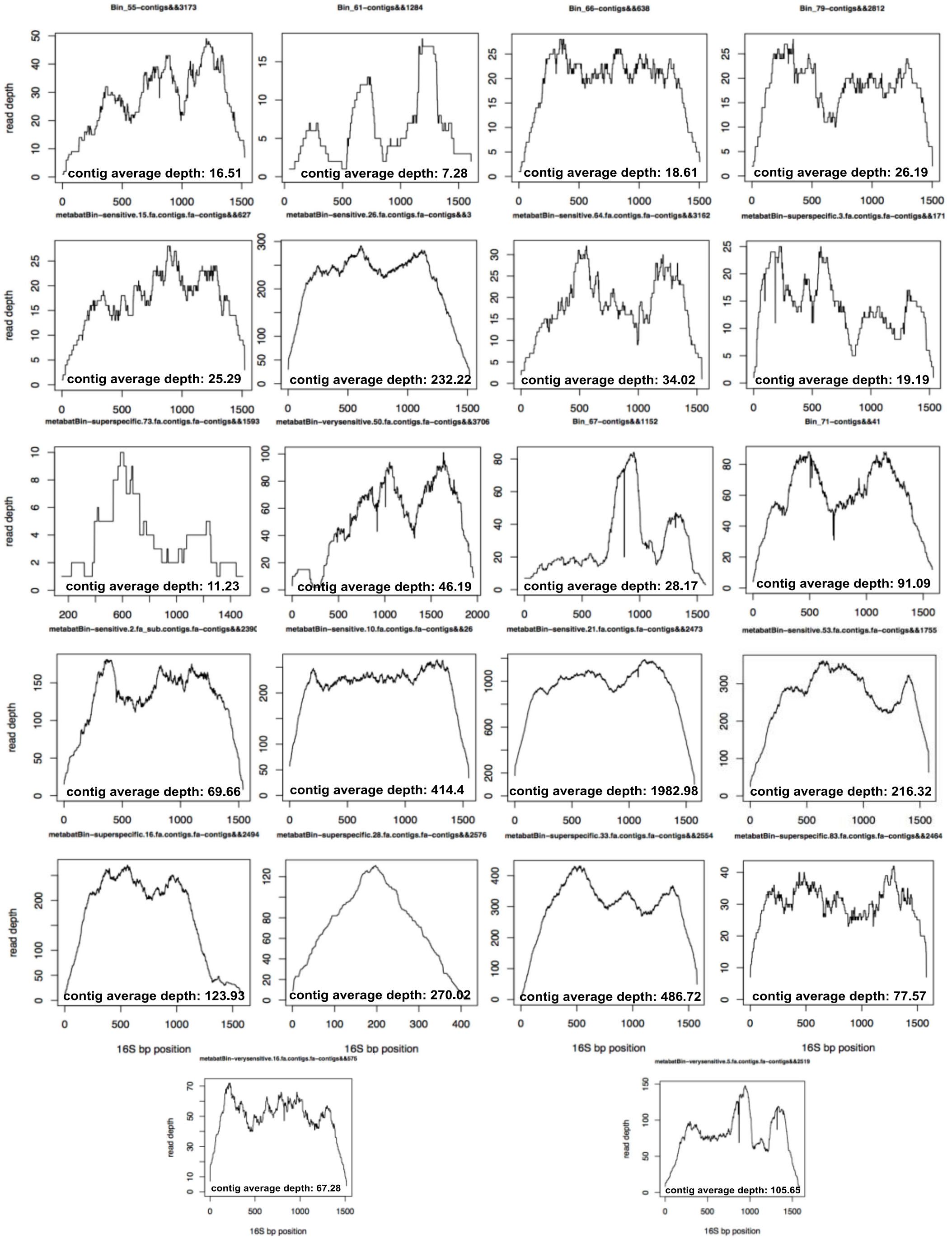

Supplemental Figure S12. Differential abundance testing using a Wald test (Pvalue=0.01) testing warm (downhole) vs. cold (wellhead) communities. This statistic is implemented on the DESeq2 package in R studio.

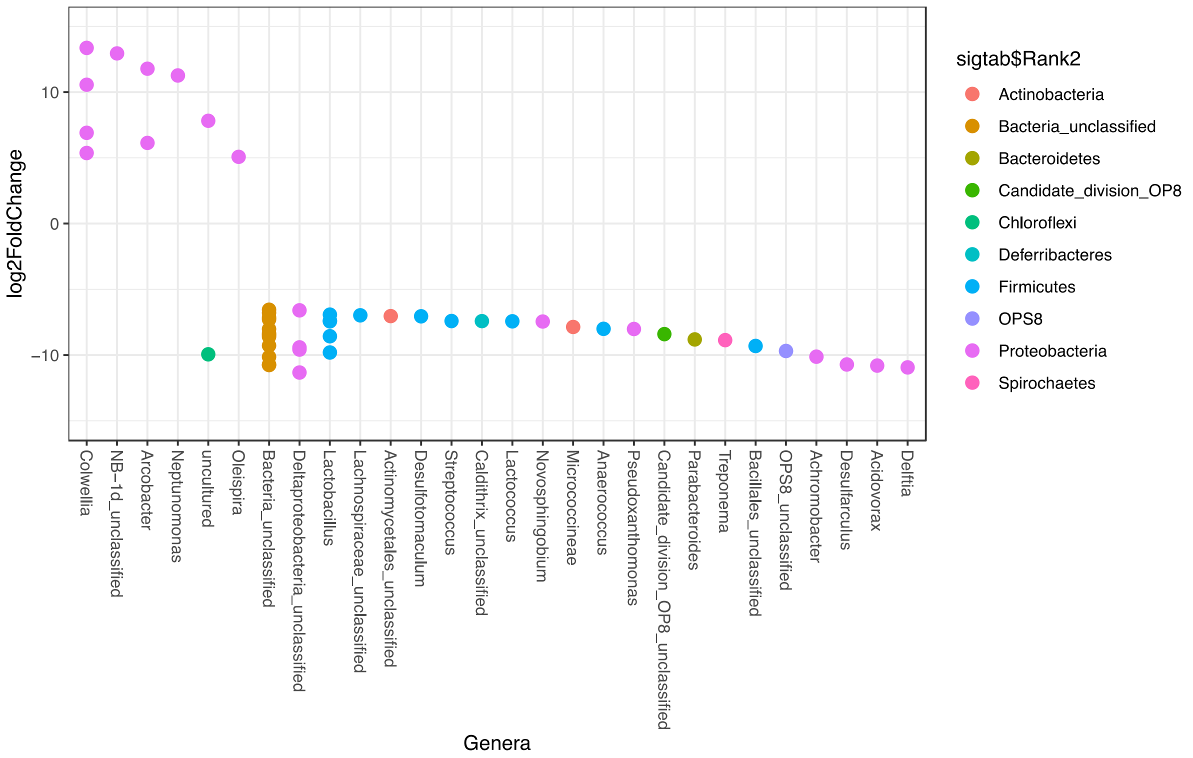

Supplemental Figure S13: Comparison of normalization strategy [A) not normalized, B) rarefied (also main text Figure 4C), and C) VST] effect on PCoA exploratory ordinations. Data rarefication and VTS transformations, were performed in R Studio using the phyloseq and DESeq2 packages.

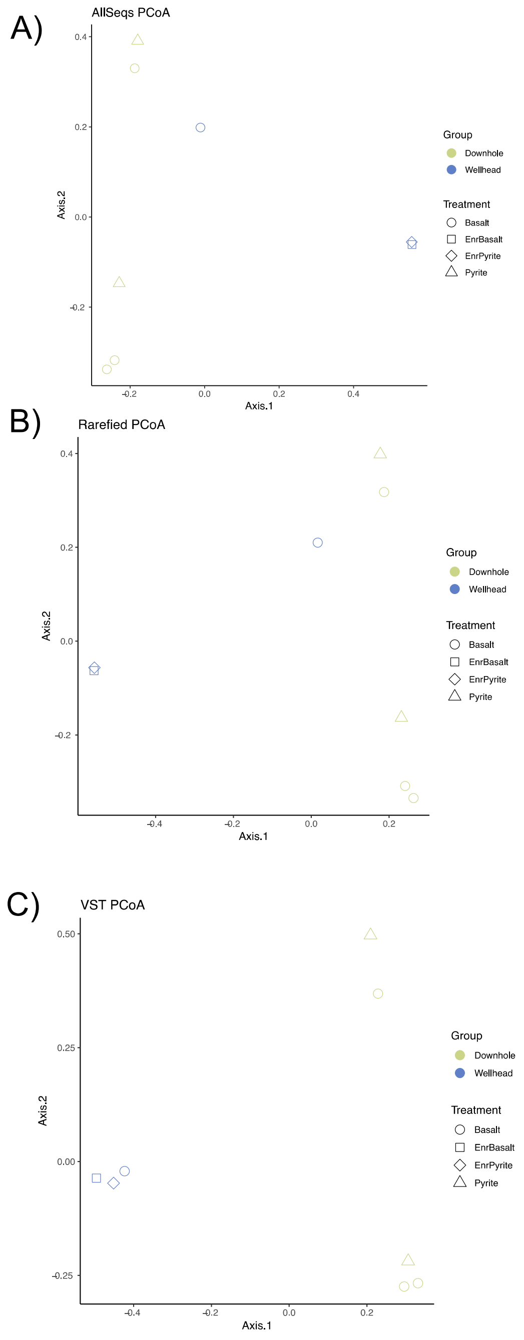

Supplemental Files captions:

Supplemental File 1:
Summary of amplicon-based recruited 16S rRNA genes from metagenome-assembled genomes (MAGs) including: reconstructed 16S rRNA gene length, PhyloSift-assigned taxonomy, closest database alignment, and percent identity/alignment length of reconstructed 16S rRNA gene sequences to their respective FLOCS-derived amplicons.

Supplemental File 2:
Tab-delimited file containing ORFs, KEGG and HMM-based functional annotations, coverage information for each ORF, and bin-OTU affiliations. These sequences represent all of the metagenomic data that was incorporated into this study.

Supplementary Information Works Cited:

1. Orcutt B, Wheat CG, Edwards KJ. Subseafloor Ocean Crust Microbial Observatories: Development of FLOCS (FLow-through Osmo Colonization System) and Evaluation of Borehole Construction Materials. Geomicrobiology Journal. 2010;27(2):143-57.
2. Jannasch HW, Wheat, C.G., Plant, J.N., Kastner, M., and Stakes, D.S. Continuous chemical monitoring with osmotically pumped water samplers: OsmoSampler design and applications. Limnology and Oceanography-Methods. 2004;2:102-13.
3. Wheat CG, Jannasch HW, Kastner M, Hulme S, Cowen J, Edwards KJ, et al. Fluid sampling from oceanic borehole observatories: design and methods for CORK activities (1990-2010). Proceedings of the Integrated Ocean Drilling Program. 2011;327(109).
4. Ramírez GA, Hoffman CL, Lee MD, Lesniewski RA, Barco RA, Garber A, et al. Assessing marine microbial induced corrosion at Santa Catalina Island, California. Frontiers in Microbiology. 2016;7(1679).
5. Orcutt BN, Bach W, Becker K, Fisher AT, Hentscher M, Toner BM, et al. Colonization of subsurface microbial observatories deployed in young ocean crust. ISME J. 2011;5(4):692-703.
6. Baquiran JP, Ramírez GA, Haddad AG, Toner BM, Hulme S, Wheat CG, et al. Temperature and Redox Effect on Mineral Colonization in Juan de Fuca Ridge Flank Subsurface Crustal Fluids. Front Microbiol. 2016;7:396.
7. Wheat CG, Jannasch HW, Fisher AT, Becker K, Sharkey J, Hulme S. Subseafloor seawater-basalt-microbe reactions: Continuous sampling of borehole fluids in a ridge flank environment. Geochemistry, Geophysics, Geosystems. 2010;11(7):1-18.
8. Reysenbach A, Wickham G, Pace N. Phylogenetic analysis fo the hyperthermophilic pink filament community in Octopus Spring, Yellowstone National Park. Appl Environ Microbiol. 1994;60:2113-9.
9. Daims H, Brühl A, Amann R, Schleifer KH, Wagner M. The Domain-specific Probe EUB338 is Insufficient for the Detection of all Bacteria: Development and Evaluation of a more Comprehensive Probe Set. . Syst Appl Microbiol. 1993(22):434-44.
10. Kozich JJ, Westcott SL, Baxter NT, Highlander SK, Schloss PD. Development of a Dual-Index Sequencing Strategy and Curation Pipeline for Analyzing Amplicon Sequence Data on the MiSeq Illumina Sequencing Platform. Applied and Environmental Microbiology. 2013;79(17):5112-20.
11. Yarza P, Ludwig W, Euzeby J, Amann R, Schleifer KH, Glockner FO, et al. Update of the All-Species Living Tree Project based on 16S and 23S rRNA sequence analyses. Syst Appl Microbiol. 2010;33(6):291-9.
12. Edgar RC, Haas BJ, Clemente JC, Quince C, Knight R. UCHIME improves sensitivity and speed of chimera detection. Bioinformatics. 2011;27(16):2194-200.
13. Quast C, Pruesse E, Yilmaz P, Gerken J, Schweer T, Yarza P, et al. The SILVA ribosomal RNA gene database project: improved data processing and web-based tools. Nucleic Acids Res. 2013;41(Database issue):D590-6.
14. Racine JS. RStudio: A Platform-Independent IDE for R and Sweave. Journal of Applied Econometrics. 2012;27(1):167-72.
15. Oksanen J, Blanchet FG, Kindt R, Legendre P, Minchin PR, O'Hara RB, et al. vegan: Community Ecology Package. R Package Version 2.2-1. Available online at: http://cranr-projectorg/package=vegan. 2015.
16. McMurdie PJ, Holmes S. phyloseq: an R package for reproducible interactive analysis and graphics of microbiome census data. PLoS One. 2013;8(4):e61217.

17. Tully BJ, Wheat CG, Glazer BT, Huber JA. A dynamic microbial community with high functional redundancy inhabits the cold, oxic subseafloor aquifer. ISME J. 2018;12(1):1-16.
18. Bolger AM, Lohse M, Usadel B. Trimmomatic: a flexible trimmer for Illumina sequence data. Bioinformatics. 2014;30(15):2114-20.
19. Magoc T, Salzberg SL. FLASH: fast length adjustment of short reads to improve genome assemblies. Bioinformatics. 2011;27(21):2957-63.
20. Bankevich A, Nurk S, Antipov D, Gurevich AA, Dvorkin M, Kulikov AS, et al. SPAdes: a new genome assembly algorithm and its applications to single-cell sequencing. J Comput Biol. 2012;19(5):455-77.
21. Sommer DD, Delcher AL, Salzberg SL, Pop M. Minimus: a fast, lightweight genome assembler. BMC Bioinformatics. 2007;8:64.
22. Langmead B, Salzberg SL. Fast gapped-read alignment with Bowtie 2. Nat Methods. 2012;9(4):357-9.
23. Li H, Handsaker B, Wysoker A, Fennell T, Ruan J, Homer N, et al. The Sequence Alignment/Map format and SAMtools. Bioinformatics. 2009;25(16):2078-9.
24. Kang DD, Froula J, Egan R, Wang Z. MetaBAT, an efficient tool for accurately reconstructing single genomes from complex microbial communities. PeerJ. 2015;3:e1165.
25. Sieber CMK, Probst AJ, Sharrar A, Thomas BC, Hess M, Tringe SG, et al. Recovery of genomes from metagenomes via a dereplication, aggregation and scoring strategy. Nature Microbiology. 2018.
26. Eren AM, Esen OC, Quince C, Vineis JH, Morrison HG, Sogin ML, et al. Anvi'o: an advanced analysis and visualization platform for 'omics data. PeerJ. 2015;3:e1319.
27. Parks DH, Imelfort M, Skennerton C, Hugenholtz P, Tyson GW. CheckM: assessing the quality of microbial genomes recovered from isolates, single cells, and metagenomes. Genome Res. 2015.
28. Kopylova E, Noe L, Touzet H. SortMeRNA: fast and accurate filtering of ribosomal RNAs in metatranscriptomic data. Bioinformatics. 2012;28(24):3211-7.
29. Darling AE, Jospin G, Lowe E, Matsen IV FA, Bik HM, Eisen JA. PhyloSift: phylogenetic analysis of genomes and metagenomes. PeerJ. 2014;2:e243.
30. Anantharaman K, Brown CT, Hug LA, Sharon I, Castelle CJ, Probst AJ, et al. Thousands of microbial genomes shed light on interconnected biogeochemical processes in an aquifer system. Nat Commun. 2016;7:13219.
31. Edgar RC. MUSCLE: multiple sequence alignment with high accuracy and high throughput. Nucleic Acids Res. 2004;32(5):1792-7.
32. Fisher AT, Wheat CG, Becker K, Davis EE, Jannasch H, Schroeder D, et al. Scientific and technical design and deployment of long-term subseafloor observatories for hydrogeologic and related experiments, IODP Expedition 301, eastern flank of Juan de Fuca Ridge. 2005;301.
33. Expedition 327 Scientists (2011), Site U1362, in Proceedings of IODP, vol. 327, edited by A. T. Fisher, T. Tsuji, K. Petronotis, et al., Integrated Ocean Drilling Program Management International, Inc., Tokyo. Scientific Drilling. 2012(13, April 2012):4-8.
